# Demographic and zoological drivers of infectome diversity in companion cats with ascites

**DOI:** 10.1128/msystems.00636-24

**Published:** 2024-08-09

**Authors:** Yankuo Sun, Jiabao Xing, Sijia Xu, Yue Li, Jianhao Zhong, Han Gao, Song Cheng, Jun Dong, Tianyou Zhang, Gang Lu, Guy Baele, Guihong Zhang

**Affiliations:** 1 Key Laboratory of Zoonosis Prevention and Control of Guangdong Province, College of Veterinary Medicine, South China Agricultural University, Guangzhou, China; 2 Maoming Branch, Guangdong Laboratory for Lingnan Modern Agriculture, Maoming, China; 3 Guangdong Laboratory for Lingnan Modern Agriculture, Guangzhou, China; 4 CAU Dong Jun laboratory, Guangzhou, China, Guangzhou, China; 5 Guangzhou Chimelong Safari Park, Guangzhou, China; 6 Department of Microbiology, Immunology and Transplantation, Rega Institute, KU Leuven, Leuven, Belgium; The University of Maine, Orono, Maine, USA

**Keywords:** infectome, meta-transcriptomics, demography, zoology, infectious diseases, cats

## Abstract

Cats (*Felidae*) have become an integral part of many households. However, our understanding of the full spectrum of pathogens affecting cats (referred to as the infectome) is limited, mainly due to the inadequacy of commonly used diagnostic tools in capturing the complete diversity of potential pathogens and the prevalence of pathogen co-infections. In this study, we employed a meta-transcriptomic approach to simultaneously characterize the infectome contributing to different disease syndromes and to investigate spatial, demographic, and ecological factors influencing pathogen diversity and community composition in a cohort of 27 hospitalized cats and seven stray cats. We identified 15 species of pathogens, with *Candidatus Rickettsia tarasevichiae* and *Tritrichomonas foetus* representing potential spillover risks. Importantly, although most cases of ascites hyperplasia were explained by coinfection with multiple pathogens, we identified the potential novel clinical outcomes of *M. aubagnense* infection among cats. We demonstrated that the increase in infectome diversity can be explained by a variety of predictors including age growth, temperature increase, and a higher proportion of females, with age growth presenting the strongest effect. Fine-scale analysis indicated that a higher diversity of infectomes were harbored in young cats rather than adult ones. Our results demonstrated that most feline diseases are better explained by the presence of virus–bacteria or virus–virus coinfection. This study serves as a timely endorsement for clinical diagnosis by vets to consider the cause of a disease based on a panel of cryptical co-infecting pathogens rather than on individual infectious agents.

**IMPORTANCE:**

Frequent studies reported the risks of cats as an intermediate host of zoonotic pathogens (e.g., SARS-CoV-2). Cats have a physically close interaction with their owners through activities like petting, kissing, and being licked on the cheek and hands. However, there are still limited studies that systematically investigate the infectome structure of cats. In this study, we employed a meta-transcriptomics approach to characterize 15 species of pathogens in cats, with *Candidatus Rickettsia tarasevichiae* first characterizing infection in diseased cats. Most feline diseases were better explained by the presence of virus–bacteria or virus–virus coinfection. The increase in infectome diversity could be influenced by a variety of predictors including age growth, temperature increase, and a higher proportion of females. A higher diversity of pathogens was harbored in young cats rather than adults. Importantly, we showed the value of linking the modern influx of meta-transcriptomics with comparative ecology and demography and of utilizing it to affirm that ecological and demographic variations impact the total infectome.

## INTRODUCTION

With the acceleration of modern-day life pace and a decrease in interpersonal interactions, pet ownership has become increasingly common (1). Domestic cat (*Felis catus*) is now considered an integral part of the family unit by many individuals. Recent research suggests a complex interplay between cats and their owners, with many people experiencing significant improvements in psychological wellbeing through the ownership and physical interaction with companion animals, such as petting, kissing, and being licked on the cheek and hands (2–8). Despite the psychological importance of cats in the family unit, there is a lack of systematic studies focusing on the risks of infectious diseases that affect cat and human health. Although it is now relatively easy to genetically characterize the feline virome and discover new virus sequences in cats, most studies provide only a limited understanding of the circulating viral genomic diversity in cats due to infrequent, nonsystematic, and spatially limited sampling of target species (9–15) . Consequently, less attention has been paid to investigating the simultaneous presence and dynamics of pathogens in cat populations. Moreover, the reasons for the presence of certain viruses in some cat species or populations at certain geographic sites but not in others remain unclear. In addition to the cat virome, the interactions between cats and humans present a potential pathway for zoonotic pathogens to spread to caregivers (16). For instance, in a study assessing SARS-CoV-2 infection status from 919 companion animals in Europe, although none of the animals tested positive by the PCR, 5.8% of cats had measurable SARS-CoV-2 neutralizing antibody titers (17). A similar study in Thailand subsequently pointed out that SARS-CoV-2 RNA was present in 19 cats, suggesting suspected episodes of human infection from cats that were initially infected through contact with infected humans (18, 19).

Recent molecular and serological investigations of cat viral infectious diseases in China from 2016 to 2019 have shown that most of cats (approximate 80%) tested positive for at least one virus (20). Within these, FHV-1 and FPV showed significant seasonal prevalence. In addition to this, genomic epidemiological investigation suggests that different genotypes of feline calicivirus (FCV) circulated regionally in Kunshan (China) (21). While viral discovery studies provide valuable insights into the evolutionary history and epidemiology of pathogens, they offer a limited understanding of the factors shaping the cat pathogen spectrum (22, 23). To gain a better understanding of microbial dynamics among cat populations, it is important to move beyond descriptive host–pathogen associations and strive for a mechanistic understanding of when and where infectious pathogens are transmitted, as well as how the entire pathogen community (infectome) is influenced by the environment and local host communities (24, 25). For instance, changes in parasite richness in wild animals have been linked to habitat loss and fragmentation, indicating that anthropogenic alterations in host species composition and population densities can directly impact parasite community compositions (26–31). Similarly, anthropogenic land-use changes have been observed to influence virus community compositions, suggesting their role as a key determinant of host viromes (32–35). These findings emphasize the importance of studying community traits, such as parasite richness, to understand and predict zoonotic risks spatially and temporally.

However, a major challenge in characterizing unbiased microbial communities is the lack of an approach to acquire all genetic information within a single specimen. Previous methodologies were often based on removing as much nucleic acids outside viral particles as possible by filtering, centrifugation, lysis, and nuclease treatment, although this seldom results in a complete depletion of host RNA (36–38). In contrast, in meta-transcriptomics, total RNA (i.e., the transcriptome) is directly extracted from untreated homogenates and used for library preparation without filtering and nuclease digestion steps. A key advantage of this over other diagnostic techniques is that it has the potential to detect, in an unbiased fashion, any pathogen that produces an RNA molecule (DNA viruses, bacteria, fungi, and eukaryotes), as well as the obvious case of RNA viruses. Hence, with analysis of appropriate tissues, meta-transcriptomics may provide a one-stop diagnostic shop. Another benefit of meta-transcriptomics is that it provides an organized way to quantify each virus present in a sample. Specifically, the percentage of reads that map to a particular virus genome is a good indication of virus abundance, especially in the context of conserved host genes. In turn, abundance level can provide important indications of disease associations, whether viruses are segmented (such that genomic components have similar or different expression levels), and help identify those viruses that are in fact derived from other eukaryotic organisms present in the sampled host, such as in undigested food or prey, gut microflora, and parasites, or simply contamination (and the greater the virus abundance, the more likely that active viral infection has occurred in the sampled host). In addition, compared to genomic nucleic acids, the transcriptome comprises compact information that is more balanced across domains of life, thereby preventing the overdominance of genetic information from large cellular organisms.

This technique has been successful in revealing the entire pathogen spectrum—including viruses, bacteria, fungi, and parasites (i.e., total infectome)—in a single-infection case, making it a powerful tool for modern pathogen discovery efforts (39–44). For example, a recent meta-transcriptomics analysis of diseased pigs in China revealed that pig diseases were determined with multiple pathogen co-infection (43). The infectome of bronchoalveolar lavage fluid samples from Wuhan (China) before the emergence of SARS-CoV-2 depicted a stable core pathogen spectrum without the presence of SARS-CoV-2 (40). Applying meta-transcriptomic sequencing to small-scale cohorts offers a new opportunity to understand how host ecology and biogeography influence microbial diversity, especially when applied to host species occurring across different habitat types or environmental gradients.

In this study, we have employed meta-transcriptomic sequencing to systematically characterize the infectome communities in cats so that they can be characterized simultaneously in the context of specific syndromes in an unbiased manner (Table S1). A total of 34 individuals were sampled and sequenced from 10 different sites across China. Specifically, 27 clinical cases were sampled from pet hospitals with a detailed context of the specific syndrome, and seven stray cats were sampled around pet hospitals by recording the number of local stray cats through in-depth interviews. Using these data, we have (i) characterized the total pathogen community with transcriptional loads and genomic information of each case; (ii) assessed pathogen interactions, potential clinical manifestations, and epidemiological impacts; and (ii) tested how demographic and environmental factors (Table 1) influence the hospital‐level infectome community of clinical cases, and whether the population density of stray cats influences pathogen richness.

**TABLE 1:** Ecological covariates that may influence infectome diversity and community composition in hospitalized and stray cats

Hypothesized covariate	Tested response variables	Reasonable hypothesis of covariates on infectome diversity^*c*^	Reference(s)
Age	Month	↑ With increasing age, more asymptomatic infections were established	(27, 45–48)
Location	Latitude (longitude excluded due to over-correlation with latitude and precipitation; Fig. S1)	NA Latitude effects encompass a number of covariates due to the influence of physiography	(49–51)
Population density	No. of other living stray companions per hospital (5 km^2^ scale)	↑ With increase in quantity, greater microbial persistence within integral colonies and increased viral encounter probability from different individuals	(28, 29)
Sex	Females	↑ Females are more susceptible to infections due to behavior and physiology (testosterone)*^a^*	(27, 52–54)
Humidity	Mean humidity	↑ Sites with high humidity tend to be negative for pathogen retention	(55, 56)
Precipitation	Average annual rainfall	↓ Sites with high rainfall tend to be negative for pathogen retention	(57)
Temperature	Local temperature when sampling	↓ Too high a temperature may not be beneficial to pathogen retention and spreading, but positive for intrahost persistence	(57)
Living condition	Stray/hospitalized	Stray cats may harbor more pathogens or with divergent abundance compared with hospitalized cats	(1, 58)
COXI reads	COXI RPM	↓ Test if acquired host-relevant data size may take over the pathogen data size	–^*b*^
Raw reads	QC reads	↑ Test if sequencing data size may influence the pathogen data size	–

^*a*^ Elevation in testosterone levels can attenuate inflammatory responses by activating regulatory T cells, which in turn suppress Th17-type reactions, thereby exerting a modulatory effect on the immunological landscape (59).

^*b*^ “–” indicates no reference provided in this covariate.

^*c*^ "↑" and "↓" indicate that we suppose these covariates positively and negatively correlate with infectome diversity, respectively. "NA" indicates that the effects of this variable are diverse and may not be as expected.

## MATERIALS AND METHODS

### Sample collection and metadata variables

**Fig 1:**
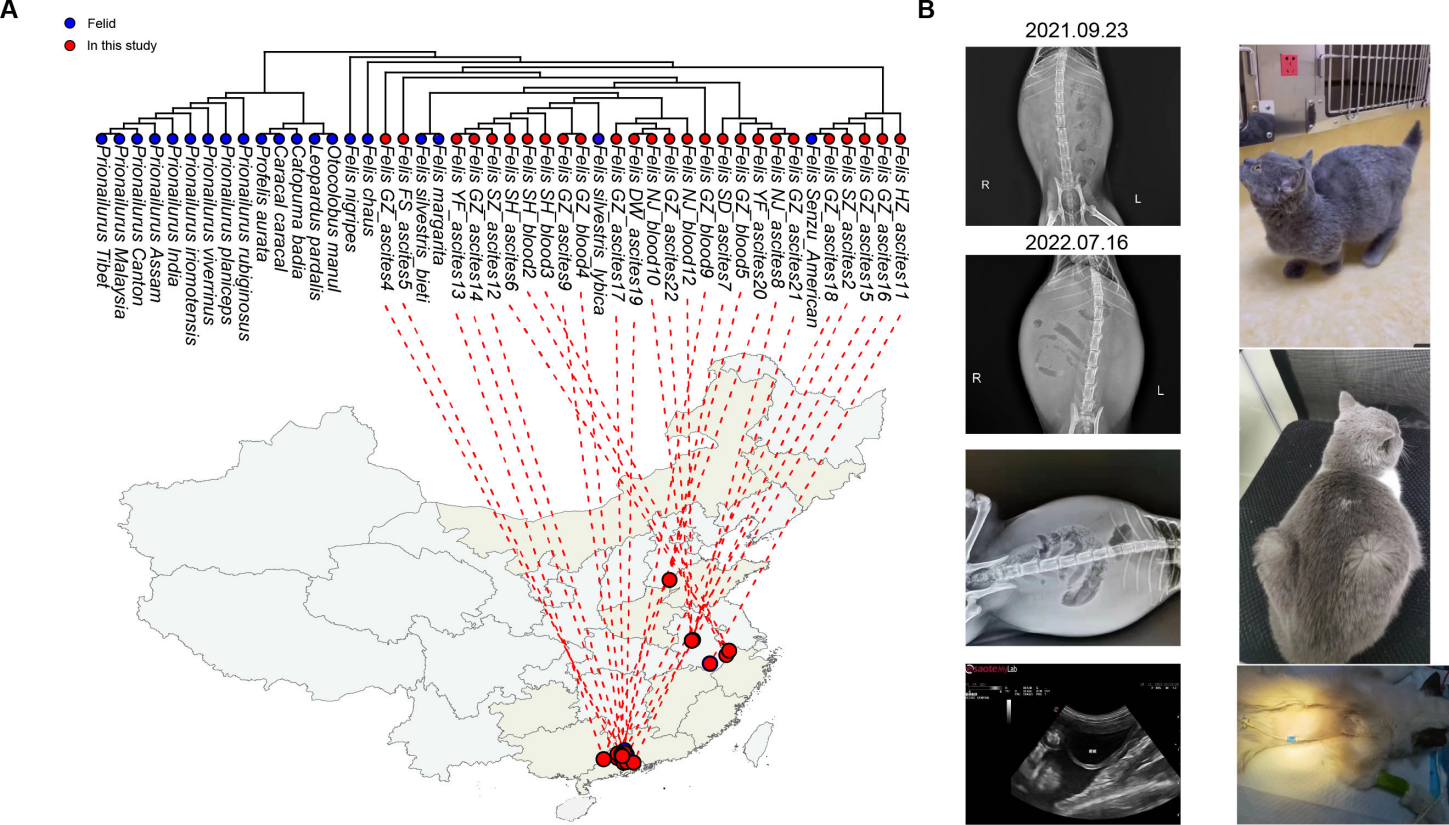
Overview of clinical cases analyzed in this study. (**A**) Phylogeny of cats based on the cox1 gene. The consensus phylogenetic tree was estimated using nucleotide sequences of the cat cox1 gene utilizing the maximum likelihood method, with 1000 replicates, with the phylogenetic tips colored by the sampling source (sequenced in this study: red, publicly available: blue). The sampled location was linked with the phylogenetic position based upon the precise location. The map was created using the R package mapdata (mapdata: Extra Map Databases, R package version 2.3, 2022). (**B**) Clinical diagnosis and treatment information of pet hospitals. We have specifically monitored a case of ascites hyperplasia whose etiology is the amino acid mutation of the spike gene (FIPV) in 1,058 and 1,060 (see Fig. S7).

From April 2022 through April 2023, we collected a total of 34 diseased cats from 10 cities in China, among which 27 had required hospitalization (Table S1). Seven of the 34 stray cats around the pet hospitals were also included in this cohort, aiming to understand the infection spectrum prevalent in the stray feline population proximal to hospitals, determine the diversity in the infectome profile of these stray cats, and compare it to hospitalized cats. Hospitalized cats included in our cohort were sampled at the initial diagnosis and admitted into the hospital, while those cats with secondary sampling or long-term hospitalization were not considered. For each case, we only collect a single sample that is unique to the neighborhood where the animal hospital is located to avoid a certain degree of empirical bias caused by subjective sampling. The severity of symptoms observed in these cases varied, ranging from mild cough to complication syndromes (i.e., fever, severe cough with expiratory dyspnea, dermatology diseases, diarrhea, and ascites hyperplasia) (Fig. 1A; Table S1). The definitions of syndrome criteria are as follows: diarrhea is defined as the passage of one or more semi-liquid or watery bowel movements, accompanied by weight loss and anorexia. Ascite hyperplasia is characterized by the presence of pleural fluid and progressive painless abdominal enlargement, which could progress to asthma or dyspnea, along with progressive muscle wasting on both sides of the spine and abdominal enlargement. Fever is defined as exceeding a temperature threshold of 39.5°C. Severe cough is described as continuous rattling sounds accompanied by back arching and chest contraction, possibly accompanied by a small amount of foam. Other possible surgically treated diseases caused by infectious pathogens, such as cutaneous diseases, acne, and dermatomycosis, were defined as dermatology diseases. However, cases involving surgical and internal medicine, such as fractures, nephrological diseases, and heart diseases, were not included in this study. Detailed symptom information for each case is provided in Table S1.

For the sampling category, we referred to the following protocol: for the individuals who showed clinical symptoms of ascites hyperplasia, we collected 1 mL of ascitic fluid using 2-mL medical injectors (22 specimens included) and transported the sample to the laboratory in a dry ice environment followed by exposure to the DNA/RNA Shield (Zymo, California, USA) mixture (1:1). For individuals experiencing other syndromes such as vomiting, fever, diarrhea, and inappetence, we collected the whole blood samples using DNA/RNA Shield Blood Collection Tube (Zymo, California, USA) to prevent blood clots, which were then transported in a dry ice environment to the laboratory (12 specimens included). The samples were stored in a −80°C refrigerator before subsequent processing. Through the sampling process, we complemented relevant environmental metadata, enabling us to explore the potential impact of covariates on pathogen diversity (for details of metadata. refer to the supplemental material). The rationale behind the selection of both sampling types arises from the need to mitigate the variability of infectome abundance that arises during the sampling process due to differential abundance profiles associated with different sample types (such as rectal swabs from diarrheal and ascitic cats or nasal swabs from respiratory tract infection cats).

### RNA extraction, library construction, and sequencing

Total RNA was extracted from the processed homogenate using the TRIzol reagent (Invitrovgen, Carlsbad, USA). No enrichment of microbial content was applied during sample processing as total transcriptomics enables to reveal the entire microbiome in a sample in an unbiased approach. RNA quality was assessed by using an Agilent 2100 Bioanalyzer (Agilent Technologies, California, USA), and the RNA quantity was quantified using Qubit 4.0 fluorometer (Invitrogen) before library construction. RNA solutions were stored at −80°C until use. Ribosomal RNA (human/mouse/rat) was removed using the Ribo-MagOff rRNA Depletion Kit (Vazyme, Nanjing, China) during the library construction, and all the 150-bp pair-end sequencing libraries were constructed using the VAHTS Universal V8 RNA-seq Library Prep Kit for Illumina (Vazyme). The library alignment’s quality and quantity were further assessed using Qubit 4.0 fluorometer (Invitrogen) and Agilent 2100 Bioanalyzer (Agilent Technologies), respectively, before sequencing. Finally, the qualified libraries were sequenced using the NovaSeq 6000 sequencer (Illumina, San Diego, USA).

### Pathogen discovery and characterization

For pathogen discovery and characterization, adapters and low-quality reads (Q30 quality) were first removed using Trimmomatic (v0.39) (60). Pathogen discovery was subjected to a pipeline analysis. All the libraries were either mapped against the non-redundant (nr) protein database using Diamond 0.9.22 with an e cutoff of 10^−5^ or were assembled *de novo* into viral contigs using MEGAHIT v1.2.8 before comparison with the nr database (61). For virus characterization, viral contigs whose amino acid content shared <90% similarity with known viruses were characterized as potential novel virus species. Viral genomes were verified by mapping each of the near-complete viral contigs to the corresponding libraries. Final coverage information in the BAM file was inspected using UGENE v40.1(62). The incomplete viral genomes were further validated using Sanger sequencing. Viral abundance information was iteratively called using Bowtie2 and calculated using the formula viral RPM abundance = total viral reads/total non-redundant reads * 1,000,000 (i.e., reads per million of total non-redundant reads [RPM]). For bacterial pathogens, MetaPhlAn2 was run to obtain the initial bacterial taxonomic profiling (63), after which their reference genomes were downloaded from NCBI GenBank as templates to call the BAM files. All contigs were further generated using BAM files and were compared with the nr database for final taxonomic classification at the species level. For fungi and parasites, relevant background mitochondrial genomes were acquired from NCBI GenBank as references to estimate the abundance. A pathogen was considered “positive” in a sample if its abundance level was greater than 1 RPM (40). To identify potential false-positives resulting from index hopping, we used a threshold of 0.1% for pathogens present in the libraries from the same sequencing lane: any read numbers < 0.1% of the most abundant library were treated as “negative,” as previously deployed (40, 44). In addition, marker genes (groEL for *Rickettsia* and 5.8S ribosomal RNA for *Tritrichomonas foetus*) were mapped to conduct phylogenetic analysis for determination of evolutionary groups (see *Phylogenetic analysis* below).

### Phylogenetic analysis

We first retrieved all the available reference sequences related to our newly identified virus species from NCBI GenBank (nucleotide database). For example, for feline coronavirus, we searched NCBI GenBank (nucleotide database) using the keywords “feline coronavirus” and downloaded the whole sequence. Duplicated sequences were eliminated in each database with a cutoff of 99% using the CD-HIT program (64). Then, we aligned the viral conserved genes or marker genes with the associated reference sequences using the progressive FFT-NS-i algorithm embedded in MAFFT v7.475. The ambiguously aligned regions were further trimmed using the trimAl algorithm and scrutinized manually (65). Afterward, maximum likelihood (ML) consensus phylogenies were estimated based on the constructed multiple sequence alignments using IQ-TREE, employing the best-matched models according to the Bayesian information criterion (BIC), as measured by ModelFinder (66). All phylogenetic consensus trees were constructed using 1,000 bootstrapped replicates, subsequently mid-point rooted and visualized using Figtree v1.4.4 (http://tree.bio.ed.ac.uk/software/figtree/) and ggtree in R v4.0.2 (67).

### Recombination analysis

We evaluated recombinant events based on Recombination Detection Program 4 (RDP4) using RDP4, Chimera, BootScan, 3Seq, GENECONV, MaxChi, and SiScanto. Furthermore, we have implemented the criterion of the highest acceptable *P*-value cutoff of 0.05 (*P* < 0.05), and it was considered a true recombination event, at least when three of the seven detection methods tested positive. Other parameters were carried out by default settings. The recombinant breakpoints were confirmed using SimPlot, with a sliding window of 200 bp (step: 20 bp). All breakpoints were further confirmed using RT-PCR and Sanger sequencing. Specifically, we first conducted recombination analyses of the FIPV genome and FeLV *env* gene. Given that all FIPV-2 strains showed recombination signals with CCoV-2, we checked the new isolates’ recombination history between FIPV-1, FIPV-1, CCoV-2, and TGEV using RDP4. For FeLV, we used FeLV-A, FeLV-B, FeLV-C, FeLV-E, FeLV-T, and enFeLV as reference groups to examine the recombination history of FeLV_53.

### Microbial diversity analysis

To infer the impact of living condition aspects to the pathogenetic microbiota composition, we estimated the α-diversity index using richness, Simpson, and Shannon indices and the β-diversity index using the Bray–Curtis dissimilarity index, using the vegan package (68). Analogously, to assess if the sampling categories may shift the pathogenetic microbiome genera structures between ascites libraries and blood libraries, we also conducted the microbial community componential analysis using α-diversity indices such as richness, Simpson, and Shannon indices and the β-diversity index using the Bray–Curtis dissimilarity index. All the statistical tests of α-diversity indices for each aspect (i.e., living conditions and sampling categories) were done using a Wilcoxon test. All the β-diversity statistics were assessed by one-factor PERMANOVA with 1,000 permutations on the Bray–Curtis matrix, using the pairwise Adonis algorithm inserted into the vegan package. Permutational tests of dispersions using the function permutest.betadisper (999 permutations, pairwise) were performed to assess whether significant effects could be influenced by differences in group dispersion. Analysis of similarity (ANOSIM) was performed using the ANOSIM function implemented in the vegan package, where *R* > 0 suggests that the intragroup distance is less than the intergroup distance, and the groupings are effective. Statistical significance of PERMANOVA results was assumed when *P* < 0.05 after the application of a Bonferroni correction (Tables S2 and S3).

### Statistical analyses of ecological and demographic correlates of infectome diversity

Generalized linear models with a Gaussian distribution were employed to identify demographic and environmental correlates of infectome diversity (measured by the Shannon index) (Table 1). All relevant environmental and demographic data are detailed in the supplementl material and deposited in Table S5. Prior to undertaking GLM analyses, Pearson’s correlation coefficients between these variables were calculated and visualized for subsequent model selection (5). Submodels were built, which excluded explanatory variables with a Pearson correlation coefficient *r* > 0.7 (Fig. S1), to aid in addressing multicollinearity, reduce overfitting, improve interpretability, and promote model simplicity. Submodels were compared with the Akaike’s information criterion corrected for a smaller sample size (AICc). Finally, we set age, COXI reads, temperature, precipitation, humidity, sex, living conditions (stray or domesticated), latitude, and raw reads as proxy predictors. Model‐averaged effect sizes and 95% confidence intervals (CIs) were calculated for each explanatory variable using the set of GLMs in which the cumulative Akaike weight summed to 0.95 using the dredge and model.avg functions of the package mumin. Effect sizes were standardized using partial standard deviation to account for multicollinearity. Relative variable importance was calculated as the sum of Akaike weights across all submodels that included each variable. Gaussian-distributed GLMs were checked for overdispersion and the normality of residuals (Table S4). GLM analyses were visualized using jtools in R. Other statistical analyses and plots were performed in R version 4.1.1 (The R Core Team, 2021).

## RESULTS

### Meta-transcriptomics sequencing and species identification of clinical cases

From the 34 meta-transcriptomic libraries, this study generated approximately 1.2 billion clean reads after quality control and trimming, with a median value of 27,614,731 reads per library (Table S1; Fig. S2). Among these data, we identified contigs associated with the cytochrome c oxidase subunit 1 mitochondrial gene (cox1 gene). The cox1 genes obtained from the 34 sequencing libraries exhibited a high degree of similarity to each other, with nucleotide identities ranging from 97.6% to 100%, representing multispecies co-existence, such as *Felis senzu american* and *Felis silvestris lybica* (Fig. 1A).

### Overview of the total infectome among clinical cases

**Fig 2:**
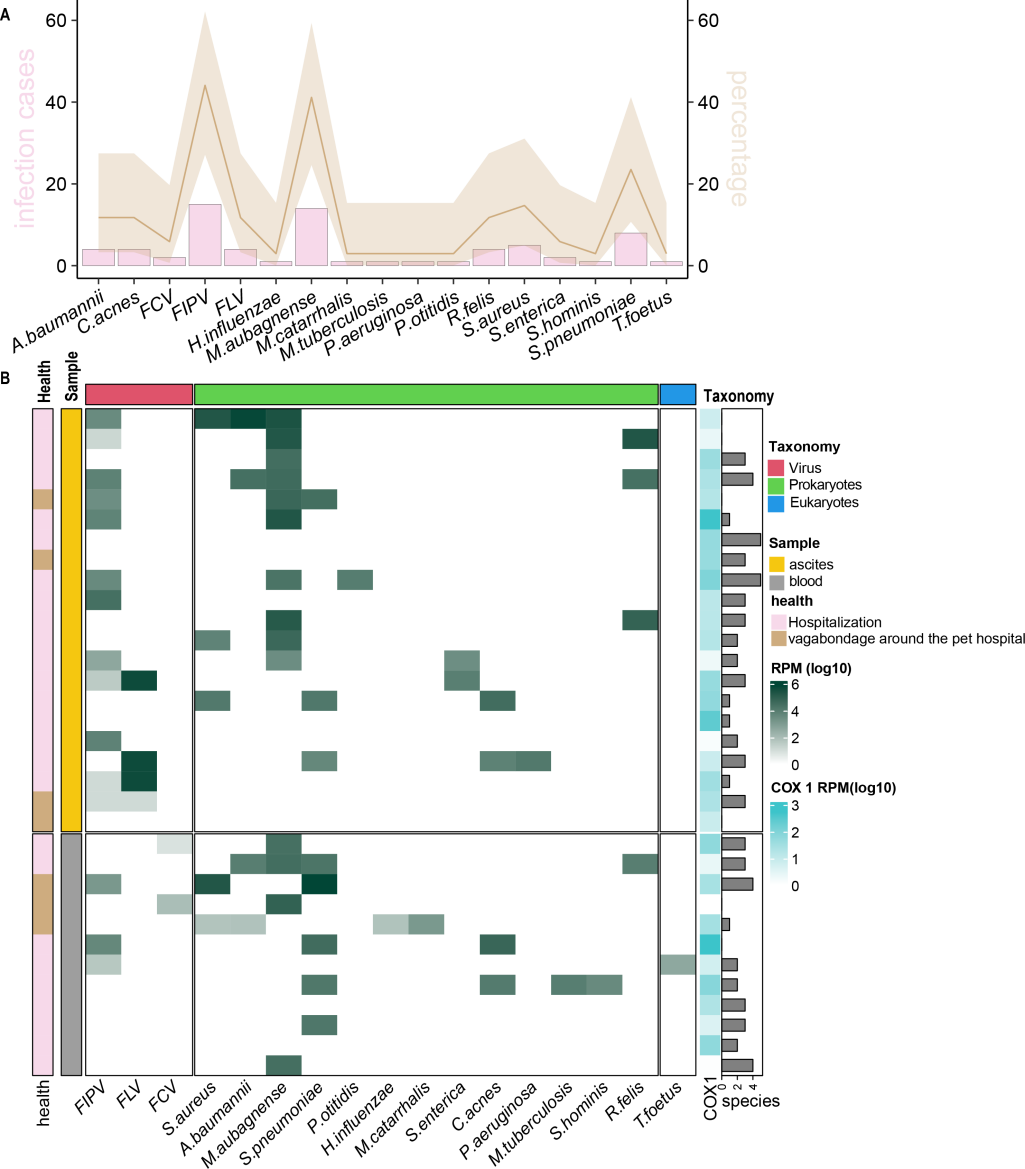
Total infectome spectrum with abundance information characterized in this study. (A) Prevalence of pathogens identified in this cohort. The bar corresponding to the left *y*-axis refers to the positive cases sequenced in this study. The line corresponding to the right *y*-axis refers to the percentage of the positive rate, with 95% CI represented by the transparent area estimated using Poisson rate estimation. (B) The relative abundance (log10-scale RPM) of specific pathogens is denoted in this heat map. The samples (*y*-axis) were divided into two groups according to the sampling categories, i.e., “ascites” and ‘blood.” They were further divided on the basis of health condition. The pathogens (*x*-axis) were first divided into two supergroups: viruses, prokaryotes, and eukaryotes.

Pathogens were identified by comparing sequencing reads directly against nonredundant protein, bacterial genome, and universal Cox1 gene databases (Fig. 2). Identifications were further confirmed using genome mapping and qPCR (or RT-qPCR) assays. In this study, we focused on (i) known pathogens, (ii) opportunistic pathogens (defined as showing pathogenicity when the host immune system is compromised or otherwise favorable), or (iii) uncharacterized viruses related to pathogens that have the potential to cause diseases in mammals. Our analysis of clinical specimens revealed the presence of various pathogens, including bacteria, viruses, and parasites. In total, three RNA viruses, 13 bacteria, and one parasite were identified in 31 out of the 34 cases in our cohort. Among these pathogens were those known to cause feline diseases, which are feline coronavirus (FCoV), feline leukemia virus (FLV), FCV, and a wide spectrum of zoonotic pathogens such as S*taphylococcus aureus* (*S. aureus*), *Acinetobacter baumannii* (*A. baumannii*), *Mycolicibacterium aubagnense* (*M. aubagnense*), *Streptococcus pneumoniae* (*S. pneumoniae*), *Pseudomonas otitidis* (*P. otitidis*), *Haemophilus influenzae* (*H. influenzae*), *Moraxella catarrhalis* (*M. catarrhalis*), *Salmonella enterica* (*S. enterica*), *Cutibacterium acnes* (*C. acnes*), *Pseudomonas aeruginosa* (*P. aeruginosa*), *Mycobacterium tuberculosis* (*M. tuberculosis*), *Staphylococcus hominis* (*S. hominis*), *Candidatus Rickettsia tarasevichiae* (*Candidatus R. tarasevichiae*), and *Tritrichomonas foetus* (*T. foetus*). Gastrointestinal pathogens, including FCoV (feline enteric coronavirus), *T. foetus*, *S. aureus*, and *S. enterica*, were also identified in our specimens. The majority of the identified pathogens were associated with respiratory infections, such as *S. pneumoniae*, *H. influenzae*, *M. catarrhalis*, and *M. tuberculosis*. Notably, we found a unique divergent infection case involving a pathogen causing infequent infection in cats, namely, *M. aubagnense*, which was first reported as the etiology of severe peritoneal effusion in humans in 2023 but was found to be highly abundant in most of the ascites samples (12 out of 17), suggesting its potential role as a neglected factor in causing peritoneal effusion in cats, similar to what has been observed in humans (69).

To assess the quantification accuracy, we compared the results obtained from both real-time qPCR (cycle threshold value [CT]) and meta-transcriptomic approaches (read per million, RPM) using a linear regression model. To do this, we designed specific real-time RT-PCR probes and primers for the most frequent infected pathogen (i.e., FIPV). The analysis revealed a strong correlation between the CT values (indicating viral copies) and the read amounts for FIPV (Fig. S3). Pearson’s correlation coefficient (*r*) was −0.76 (*P* < 0.05), indicating a reliable quantification of intrahost replicate abundance using our meta-transcriptomic approach (supplemental material; Fig. S3). Given the observed variability in pathogen abundance, we set a threshold of 1 RPM for pathogen identification, which allowed us to accurately determine the presence of pathogens in the samples (40, 43, 70).

### Complex evolutionary history of eukaryotic viruses

**Fig 3:**
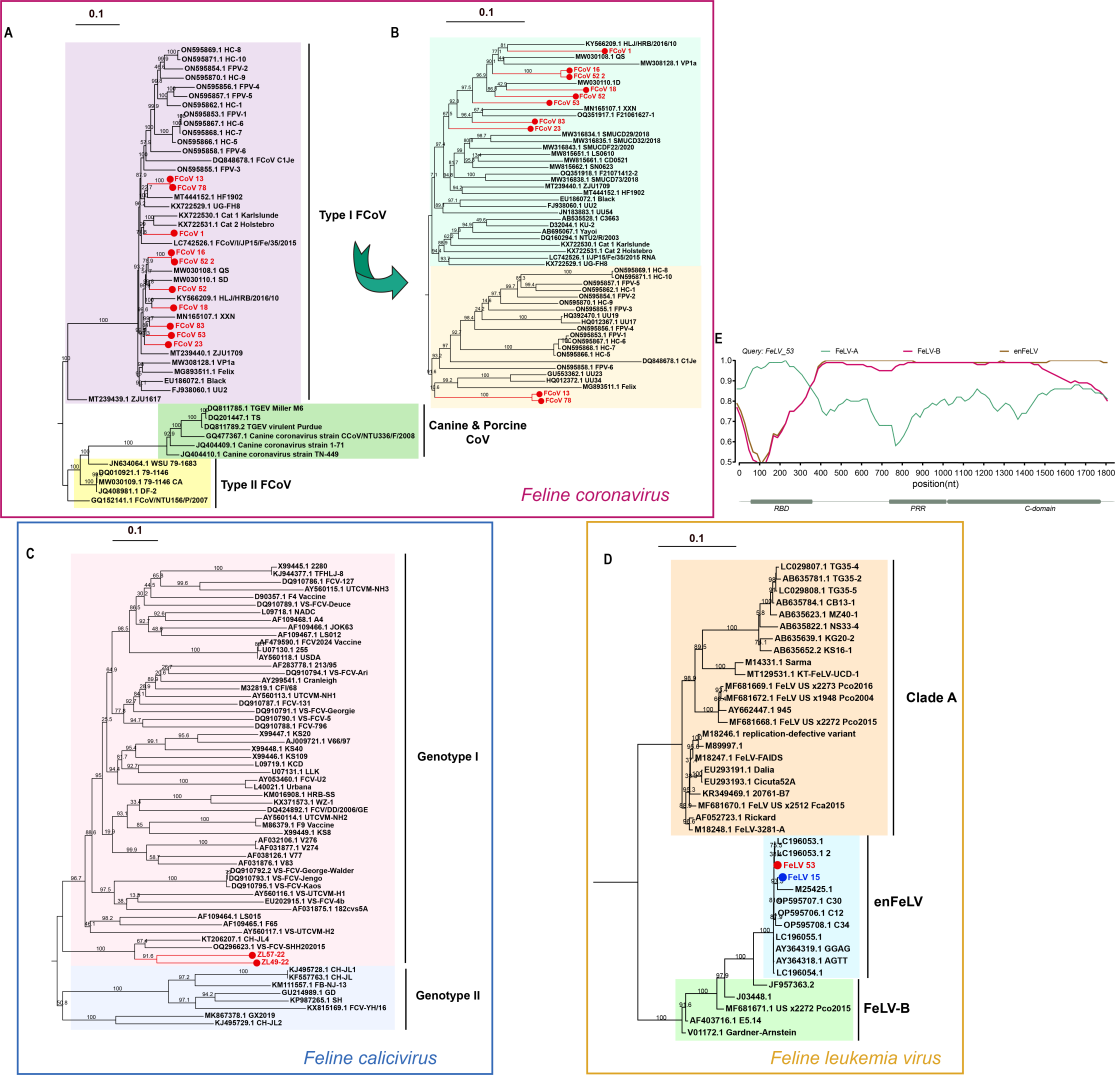
Evolutionary history of RNA viruses. Maximum-likelihood phylogenies of different RNA viruses. (A) Phylogeny of FIPV based on the complete genome. (B) Phylogeny of FIPV based on the spike gene. (C) Phylogeny of FCV based on the capsid gene. (D) Phylogeny of FeLV based on the *env* gene (red: hospitalization, blue: stray), with specific recombination shown in panel E. (E) Recombination analysis of FeLV_53 at the *env* gene.

Although we did not identify any novel viral pathogens in this study, the viruses detected exhibited significant phylogenetic diversity, indicating a complex epidemiological history within companion cats. For example, phylogenetic analysis revealed that all FCoV strains in our cohort belonged to FCoV type I, sharing nucleotide similarities of 92.6% to 99.8% (Fig. 3A). Since FCoV type II is believed to have originated from recombination events between FCoV type I and canine coronavirus (CCoV), we conducted analyses to investigate the recombination history. However, no recombination signal with either FCoV type II or CCoV was detected, supporting our phylogenetic analysis findings (Fig. S4) (71). Within the spike gene, two distinct lineages (clade 1 and clade 2) were identified, and several novel subclades were formed by our strains (Fig. 3B). We also examined specific mutations in the spike protein (M1058L and S1060A), which have been associated with feline infectious peritonitis (FIP) development in FCoV. Among our study genomes, six had the M1058L mutation, while none had the S1060A mutation (Fig. S5) (72). Phylogenetic analysis of the FCV capsid region indicated regional transmission, as our sequences clustered with other enteric sequences from east China (Fig. 3C). Although the *env* gene of FeLV 53 clustered within the enFeLV lineage, it grouped with the recombined strains, and further recombination analysis confirmed its recombination history in the receptor-binding domain, highlighting its potential risks (73).

### Genomic analysis of zoonotic pathogens

**Fig 4:**
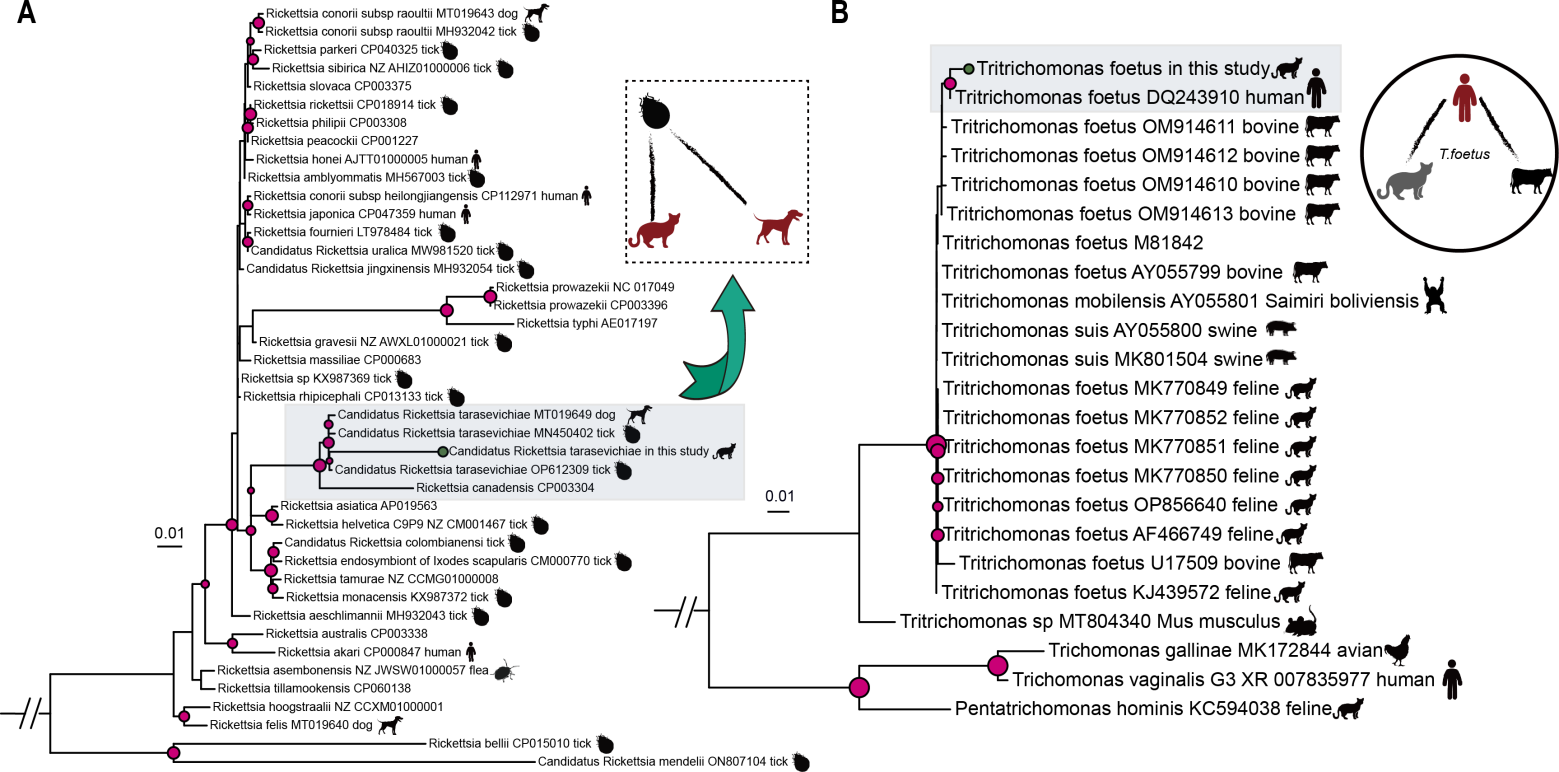
Phylogenetic analyses of potentially zoonotic pathogens. (A) Maximum-likelihood phylogeny of *rickettsia* genera based on the *groEL* gene, with the green circle at the tip indicative of identification in this study. Trees were midpoint rooted, and bootstrap values > 70% from 1,000 bootstrap replicates are linked with the gradient size of the red circle at the node. The host distributions are shown with a cartoon pattern. (B) Maximum-likelihood phylogeny of *Tritrichomonas* genera based on 5.8S ribosomal RNA, with the green circle at the tip indicative of identification in this study. Trees were midpoint rooted, and bootstrap values > 70% from 1,000 bootstrap replicates are linked with the gradient size of the red circle at the node. The host distributions are shown with a cartoon pattern.

In addition to identifying viral pathogens causing feline diseases, we also detected several bacterial pathogens with relatively high abundance through meta-transcriptomics analysis (Table S1). Among these findings, we characterized that cats served as a potential novel host for two pathogens. First, we discovered a novel tick-borne mammalian pathogen belonging to the species *Candidatus R. tarasevichiae*, with the highest amino acid similarity of 93.4% with other sequences available in GenBank at the *groEL* gene. Phylogenetic analysis showed that the *Candidatus R. tarasevichiae* in our study clustered within the well-established spotted fever group but displayed significant evolutionary divergence from strains identified in other hosts, such as dogs and ticks (Fig. 4A). This finding is noteworthy as no previous study has reported the infection of *Candidatus R. tarasevichiae* in cats. The detection of this pathogen, particularly in cases with fever and respiratory manifestations, suggests its ability for cross-species transmission (i.e., cats may serve as a novel host for infection transmission from ticks) (74, 75). We also conducted phylogenetic analyses of *T. foetus* based on the small 5.8 s subunit ribosomal RNA. Typically, all the isolates cluster according to their respective infection hosts. Interestingly, the trichomonad isolates identified in our study clustered together with the human fetal *T. foetus* isolate (DQ243910), which was isolated from bronchoalveolar lavage samples of AIDS patients, highlighting the risk of cross-species transmission (Fig. 4B) (76).

### Co-infection dynamic of different pathogens

**Fig 5:**
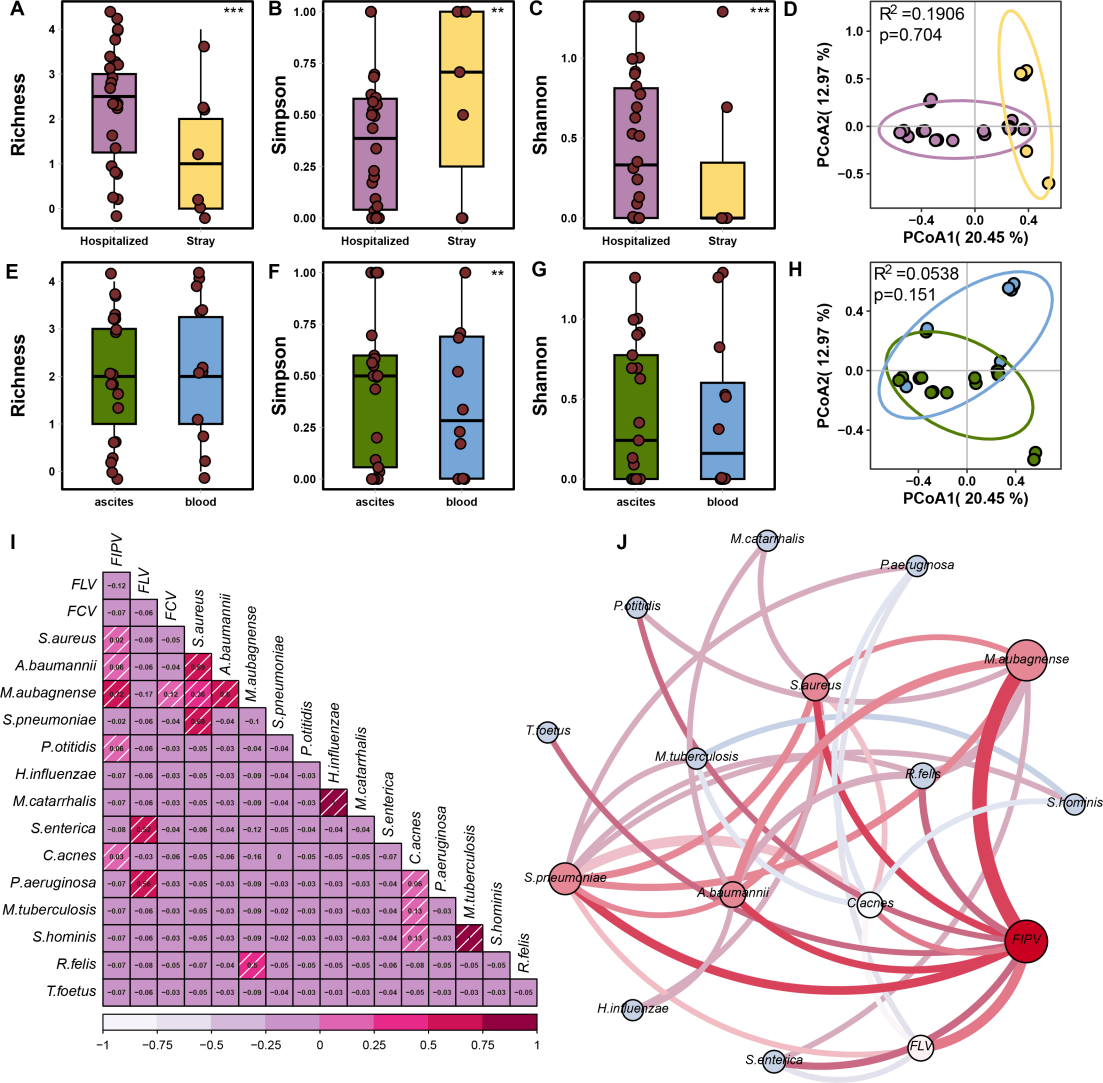
Comparisons of infectome diversity and composition metrics in our cohort. (A–D) Microbiota diversity characterization against different health conditions. (A) Richness index of pathogenetic microbiota of different health conditions. B. Simpson index of pathogenetic microbiota of different health conditions. (C) Shannon index of pathogenetic microbiota of different health conditions. (D) Beta diversity analysis of different health conditions. Principal co-ordinates analysis based on Bray–Curtis dissimilarities at the species level. Differences between the different health conditions were calculated based on the PERMANOVA test. Ellipses are at the 95% CI. (E–H) Microbiota diversity characterization against different sample types. (E) Richness index of pathogenetic microbiota of different sample types. (F) Simpson index of pathogenetic microbiota of different sample types. (G) Shannon index of pathogenetic microbiota of different sample types. (H) Beta diversity analysis of different sample types. Principal co-ordinate analysis based on Bray–Curtis dissimilarities at the species level. Differences between the different sample types were calculated based on the PERMANOVA test. Ellipses are at the 95% CI level. (G) Pearson’s correlation analysis of pairwise concurrent pathogens at abundance levels, with *P* < 0.05 shown as shading. (I) Co-infection network of important pathogens. Specifically, thickness and gradient colors (from white to red) of links of paired pathogens denoted the frequency of pairwise coinfection of two pathogens. The gradient color of nodes was proportional to the degree of prevalence rate in our study cohort. The size of each node represented the co-infection frequency with other pathogens (from white to red). Pairwise statistical tests of α-diversity indices were examined using a Wilcoxon test.

To examine the differences in pathogen composition and co-infection patterns between hospitalized cats and stray cats, we compared the infectome profiles obtained from these two groups. In hospitalized cats, the majority of cases were associated with multiple pathogens (19/26), whereas fewer cases with multiple pathogens were observed in stray cats (Fig. S6). This suggests that the infectomes of diseased cats are highly complex and cannot be fully understood using a single pathogen disease model. Moreover, we found that the abundance diversity of pathogenic microbiota was significantly higher in hospitalized cats than in stray cats (Fig. 5A through C). Principal coordinates analysis (PCoA) revealed a distinction between the infectome communities of diseased cats and stray cats (permutational multivariate analysis of variance PERMANOVA], *P* adjusted = 0.704, *R*^2^ = 0.1906, Fig. 5D). However, when considering the sample type, our results did not show a significant difference in pathogenetic communities between blood and ascites samples (Fig. 5E and G). Additionally, there was no significant variation in total infectome richness, and the infectome communities did not cluster based on sampling types (Fig. 5H). We further examined the co-infection frequency of paired pathogens. The co-occurrence networks of diseased cats indicated that most coinfection cases involved a combination of viruses and bacteria, such as FIPV and *M. aubagnense* (Pearson *r* = 0.72) or FIPV and *S. pneumoniae* (Pearson *r* = 0.02) or FCV and *M. aubagnense* (Pearson *r* = 0.12). Furthermore, most cases presented a bacteria-associated co-infection spectrum such as *M. aubagnense* and *S. aureus* (coefficient = 0.69) or *S. aureus* and *S. pneumoniae* (coefficient = 0.69, Fig. 5I and J).

### Association between clinical manifestations and pathogen co-infection

**Fig 6:**
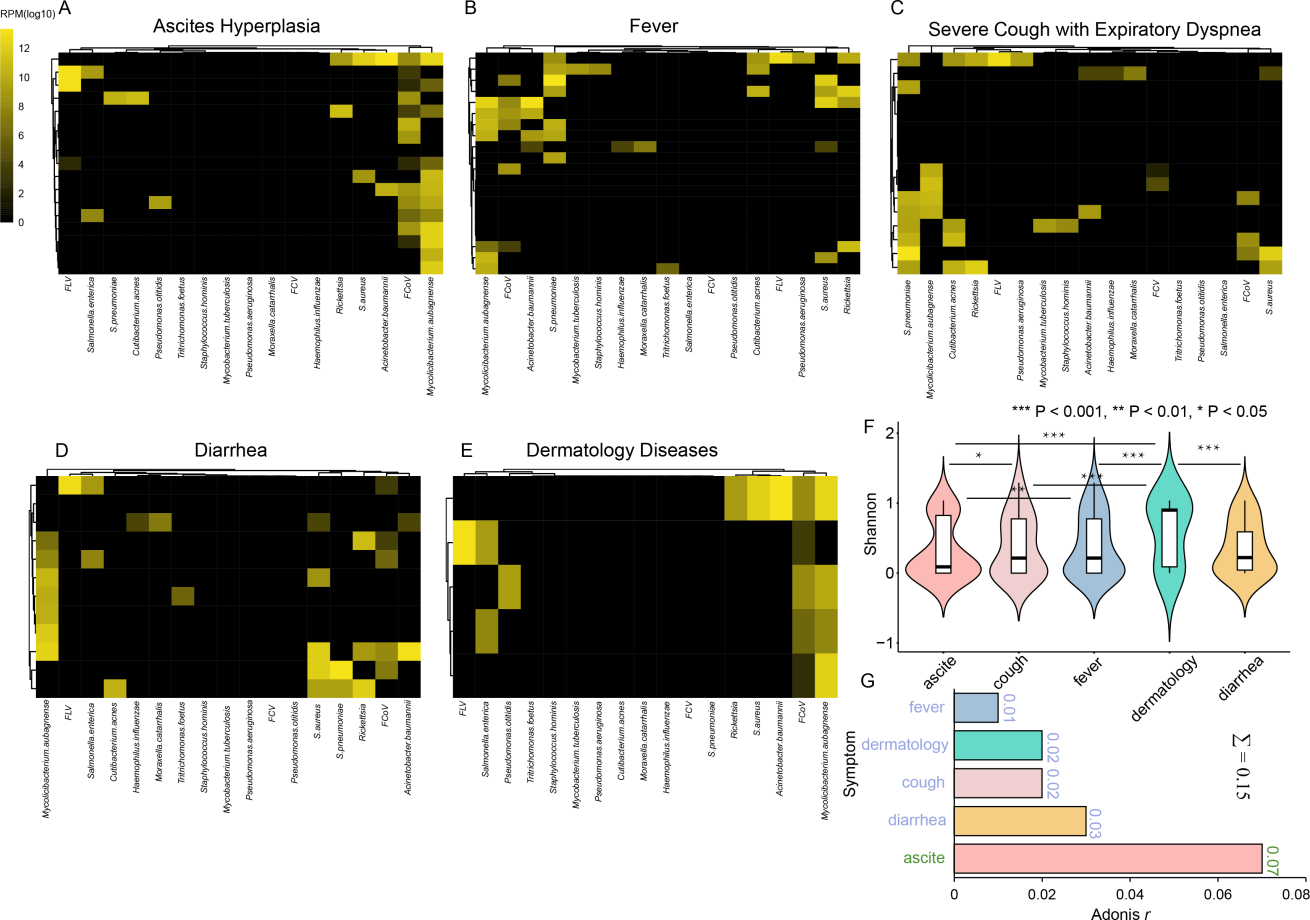
Association of pathogen infection and clinical manifestations in cats. (A–E) For each of the disease types, a heat map displays the prevalence and abundance of the pathogens identified. Disease types include general symptoms (i.e., ascites hyperplasia, fever, severe cough with expiratory dyspnea, diarrhea, and dermatology diseases). (F) Shannon index of pathogenetic microbiota of different clinical manifestations. (G) Bar plot illustrating each manifestation associated with the variation of the total infectome. Each manifestation was ranked by their collective Adonis R-squared value. Pairwise statistical tests of α-diversity indices were examined using the Wilcoxon test.

The complex nature of co-infections presents challenges in establishing definitive causal relationships between pathogens and diseases. To address this, we conducted a comprehensive analysis that considered clinical symptom results in the context of a panel of relevant pathogens with indication of abundance, rather than focusing solely on individual pathogens. In cases of ascites hyperplasia, most co-infections were associated with a high abundance of both *M. aubagnense* and FIPV. For cases presenting fever and respiratory symptoms, there was a convergence of co-presenting pathogens to some extent, including FIPV, *S. aureus*, *Rickettsia*, *A. baumannii*, and *S. pneumoniae*, with a lower abundance of FIPV observed in respiratory models. Conversely, digestive symptoms or diarrheal models often involved combinations of three or more pathogens, many of which were present in high abundance (Fig. 6A through E). In addition, the diversity difference of individual symptoms was calculated and compared using the Shannon metric, which indicated that the diversity of pathogens within the host showing ascite symptoms was significantly lower than that showing other symptoms (Fig. 6F). The proportion of variance in microbiome composition that can be explained by individual symptoms was calculated by permutational multivariate analysis of variance using distance matrices (adonis). These symptoms explained 15.3% of infectome taxonomic composition, with the largest weight from ascite symptoms, followed by diarrhea, cough, and dermatology (Fig. 6G).

### Ecological drivers of infectome community composition

**Fig 7:**
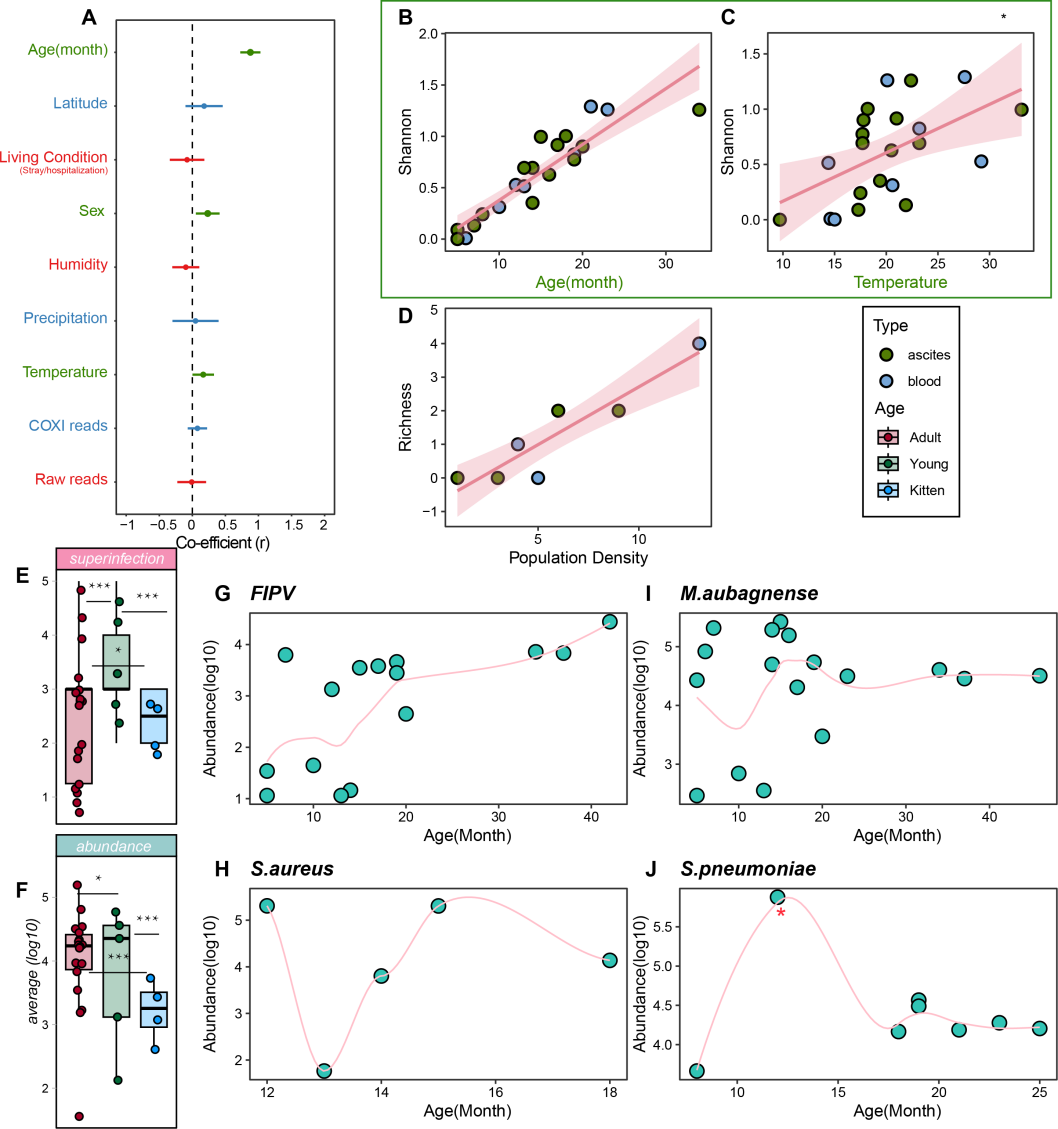
Ecological and demographic correlates of infectome diversity in our cohort. Model‐averaged relationships of demographic and ecological factors with diversity (Shannon and richness) and univariate correlations of significant factors. (A) In the model‐averaged results, effect sizes are shown for each factor across a set of GLMs with 95% CI. Factors that remained significant in the final model are shown in green. The vertical dashed line shows an effect size of 0, such that any CI overlapping the dashed line indicates a nonsignificant effect of the factor in model‐averaged results. (B and C) Shannon values are plotted for each variable (age and temperature) that was significant according to model averaging. Solid lines show GLM predictions for univariate relationships that remained significant following correction for multiple testing, with transparent shading indicating 95% CI. Points are colored according to sampling types (blue, ascites; green, blood). (D) Richness values are fit into population density of stray cats with the linear model. Solid lines show linear model predictions, with transparent shading indicating 95% CI. Points are colored according to sampling types (blue, ascites; green, blood). (E and F) Box-line plot compared (E) superinfection numbers and (F) average abundance in three groups (i.e., adult, juvenile, and kitten) as well as the *P*-values of the Wilcoxon test on the levels of each group. (G–J) Locally weighted regression (LOESS) modeling the predictions for univariate association between pathogen abundance and age using FIPV, *M. aubagnense*, *S. aureus*, and *S. pneumoniae*, with the outlier marked with an asterisk (J). Pairwise statistical tests of α-diversity indices were examined using the Wilcoxon test.

We constructed Gaussian-distributed generalized linear models (GLMs) with proxy ecological and demographic variables as predictors for diversity metrics. Submodels of GLMs were created to exclude highly correlated variables (i.e., >0.7; Fig. S1). Since multipathogen co-infection was common across the entire study cohort, we used the Shannon index as the response variable. We tested age, COXI reads, temperature, precipitation, humidity, sex, living conditions (stray or domesticated), latitude, and raw reads as proxy predictors. Variable selection on submodels revealed that COXI reads, precipitation, humidity, living conditions (stray/hospitalization), latitude, and raw reads had no significant effect on the diversity of pathogen immunity. In comparison, age, sex, and temperature showed significant effects on the diversity and abundance of the pathogen community (Fig. 7A through C), with age presenting the strongest effect (z-value = 10.58, *P*-value = 3.75E−09, and coefficient = 0.41). This suggests that host age may significantly shape the structure of the infectome, more so than other predictors (Fig. 7; Table S4). Interestingly, our linear regression model also rejected the null hypothesis that the population density of stray cats has no effect on pathogen infectome diversity, indicating that as the population density of stray cats increases, the diversity of the total infectome may also increase (Fig. 7D).

**Fig 8:**
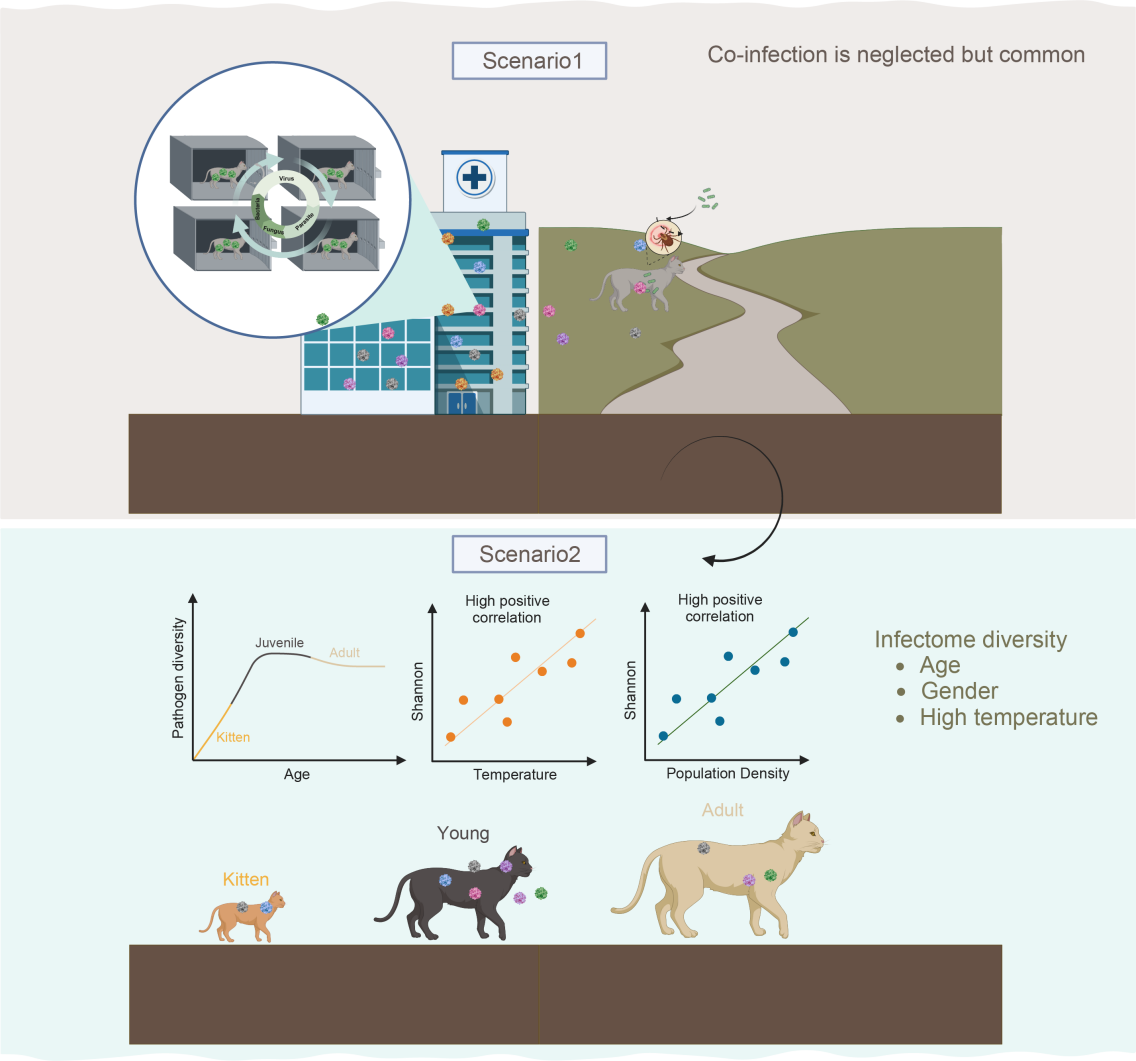
Geographical diagram to understand the ecological and demographic contribution to the clinical infectome. Two hypotheses are proposed that may explain the focal distribution of hospitalized and stray cats.

Given the strong impact of age, we conducted additional statistical analyses to evaluate the effect of host age structure on the infectome community. First, we categorized cats into three groups based on age: kittens (0–6 months), young (6–12 months), and adults (13–72 months) and compared *in vivo* pathogen diversity and abundance). Higher numbers of superinfection cases were observed among young individuals, followed by adults and kittens (Fig. 7E and F). Similarly, infection abundance was highest in kittens, followed by adults and juveniles. We also examined whether specific prokaryotic or viral pathogens (e.g., FIPV, *M. aubagnense*, *S. aureus*, and *S. pneumoniae*) differed in their abundance or prevalence according to host age using locally weighted smoothing fitting (Fig. 7G through J). We observed that the abundance of FIPV and *M. aubagnense* increased significantly during the 10–15-month period (young stage), followed by a slower increase in subsequent infection periods (adult stage). The association between pathogen abundance and age was not clearly observed for *S. aureus* and *S. pneumoniae* due to the limited number of cases available (Fig. 7H through J). These findings suggest that young cats are more likely to harbor a greater number of pathogenic taxa with higher abundance than adults and kittens (Fig. 8). In summary, these results indicate that host and ecological variation can predetermine the likelihood of infectious disease-associated infections.

## DISCUSSION

Our knowledge of the infectome of companion cats is still limited, especially when it comes to understanding the ecological factors that influence pathogen infections in these animals. The diversity and factors contributing to variation in infectome communities among populations of companion cats have been largely unexplored, despite their importance in understanding the impact of environmental changes or human activities on infectious diseases.

This study is the first epidemiological cohort research based on infectome insight. Specifically, we first determined the infectome of cats (both pet and stray) in hospitals across various geographical locations in south China. This understanding will contribute to the current epidemiological baseline of domestic cat infectome, where a broader sampling schema better characterizes the infectome of cats in the south China region. Second, we examined the potential drivers of infectomic diversity among samples across different cities. In this scenario, if sampling is performed in a singular geographic location, the inherent environmental factor variances are minimal, making it challenging to fully characterize the influences on potential infectomic diversity. Lastly, we acknowledge that the epidemiology of pet cat pathogens in a single area exhibits certain similarities and cannot adequately reflect the broader epidemiology status of domestic cats in the south China region. Based on these scenarios, we sampled diseased cats from south China rather than one single fixed-point sampling.

The deployment of meta-transcriptomic sequencing enabled us to characterize the pathogen spectrum of domestic cat populations in an unbiased manner. In total, we have acquired 15 pathogen species, comprising three RNA viruses, 13 bacteria, and one parasite. Although transcriptomic analysis of total pathogens alone is unlikely to completely reveal the clinical consequences and fine mechanisms for each pairwise occurrence of pathogens, we carefully examined the potential manifestation of pathogen interaction with different symptom models. In our study, a substantial number of the cases (64.71%, 22/34) were characterized by the presence of at least two pathogens in a single clinical case. Hence, these findings did not easily fit the paradigm of “one disease, one pathogen.” Virus–bacteria co-infection was the most frequently observed in our cohort, specific to different manifestations. In virus–bacteria co-infection cases, the highest pairwise occurrence is the co-infection of FIPV and *M. aubagnense*. Notably, *M. aubagnense* was frequently documented as the potential causative etiology of respiratory secretions and joint fluid, with a rare focus on ascites hyperplasia. However, Du et al. (69) first postulated it as the causative etiology of severe peritoneal effusion in a patient in Guangxi (69). Hence, we further examined this scenario with a single-pathogen model, which showed that in cases of ascites, 70.59% (12/17) of cats were found to be infected with *M. aubagnense*, corroborating that the hypothesis of *M. aubagnense* as the potential etiology of ascite hyperplasia is not limited to humans but occurs even among cats. Generally, an impaired host immune system, damaged epithelial barrier, and excessive inflammatory response can result in complex infection scenarios and severe clinical outcomes (23). At the very least, the strong correlation between FIPV and *M. aubagnense* may act synergistically, resulting in changes in pathogen activity, exacerbating the clinical manifestations, or lead to longer disease duration than that of individual infections alone.

Of particular interest is the identification of *Candidatus R. tarasevichiae* infection in cats. *Candidatus R. tarasevichiae* has been previously recognized as a tick-borne pathogen primarily associated with ticks and rodents as vector hosts (77, 78). However, there have been reports of meningitis-like manifestations in fatal cases of rickettsiosis caused by *Candidatus R. tarasevichiae* in China (75, 79). Additionally, a fatal infection in a 4-year-old girl with typical symptoms of tick-borne rickettsiosis (fever, rash, eschar at the site of the tick bite, and myalgia) and meningeal involvement was documented, revealing a co-infection with mixed *R. sibirica* and *Candidatus R. tarasevichiae* in Russia (80). While these studies have expanded our understanding of the host range of *Candidatus R. tarasevichiae*, no previous study has reported cats as the potential host for *Candidatus R. tarasevichiae*. In our study, a cat (no. L01_19) infected with *Candidatus R. tarasevichiae* exhibited severe fever, cough with expiratory dyspnea, and diarrhea. Although we failed to learn the potential source of infection, this finding also expanded the potential host spectrum of *Candidatus R. tarasevichiae*. Another intriguing observation is the characterization of *T. foetus*, which showed a closer phylogenetic relationship to the human sequence. This finding suggests a mysterious connection between the two strains. Given that companion cats frequently interact with their owners, there is a particular concern regarding that cats may represent an additional reservoir host that could impact human infection rates via dilution or amplification effects (81). Therefore, it is crucial to conduct thorough and periodic investigations of infectious pathogens in companion animals and local tick populations to mitigate potential zoonotic threats.

Although our study revealed significant variation in pathogen communities among populations of the same species, our GLM analysis rejected the null hypothesis that the similarity of pathogenic microbiota communities is solely driven by spatial proximity or symptom-clustered disease models. Instead, our modeling analysis supported the hypothesis that gender, temperature, and age, rather than geographical variation or sequencing capacity, influence the diversity of the pathogen spectrum. Specifically, we observed a significant correlation between age and pathogen diversity (*r* = 0.41). This suggested that older cats are more likely to harbor a greater diversity or higher abundance of pathogens. However, when we grouped cases based on three major age periods (kitten, adult, and juvenile) to examine the effect of age, we found that juveniles, rather than adults, retained the highest pathogen community diversity regardless of taxa or abundance. Further analysis focusing on individual pathogens confirmed our findings that cats aged between 10 and 20 months were more likely to harbor pathogens such as FIPV and *M. aubagnense*. After 20 months, the pathogen burden seemed to decline drastically (Fig. 7G through J). We postulated that this age-based difference in pathogen shedding may be attributed to age-related variations in innate immunity or possibly adaptive immunity specific to these taxa. Previous studies have shown that certain populations of swans maintain long-lasting immune responses to avian influenza virus, making adult swans less susceptible to influenza-associated mortality (82). Indeed, this is not the only case revealing the effect of age against pathogen infection. Hill et al. (22) also showed that perturbations that affect population age structures of wildlife could alter pathogen transmission dynamics (83). Similarly, a study suggested that colonies with a higher proportion of juveniles consistently had more diverse fecal viral communities (24). To the best of our knowledge, our study is the first to demonstrate that age heterogeneity may influence infectome diversity. Therefore, this host-level effect may be driven by age-related demographic factors such as mobility, diet, or maternal stress or differential exposure rates to pathogens among different age classes. For example, independent young cats may have increased exposure to many pathogens due to their higher mobility. However, their immune levels may not undergo corresponding enhancement, thereby increasing the likelihood of pathogenic infections. Another host factor that significantly influences pathogen gain is gender. A number of studies have suggested the association between gender and parasites such as cestode and nematode, with only a few rare studies explicitly focusing on total infectious diseases (52–54, 84). Conversely, female cats were observed to have more diverse pathogen infections. We hypothesized this might be linked to the distinct behavior and physiology of female cats. For example, risks of maternal complications (infection, urinary retention, hematoma, or ruptured sutures) in the postpartum period increase with duration of the second stage of labor also after accounting for maternal, pregnancy, and delivery characteristics (85). However, this scenario should be interpreted cautiously, as these observations are totally human-based, with no direct clinical-based evidence reflecting on cats explicitly. In addition to host variables, we also examined ecological factors that contribute to predicting pathogen diversity. Our findings suggest that warmer environmental conditions increase pathogen transmission (and/or environmental persistence) and lead to a higher number of pathogen infections. In humans, temperature has been strongly associated with bacterial infections, directly transmitted viruses, and helminth infections on a global scale (55).

Our study has several limitations. (i) While sequencing to high depth allowed us to recover the total microbiome and detect how ecological and host conditions shaped pathogen prevalence and abundance, our characterization was nevertheless dominated by bacteria rather than viruses. As mentioned before, this might be the nature of differences in ethology, living conditions, or human intervention in divergent animal models. (ii) Although we did test the ecological function and evolutionary details of variation in bacteria, pathogenic viruses, and parasite in this study, it is possible that a more generalized microbiota deputation such as total virome (including prokaryotic and eukaryotic) may equally hold ecological importance. Although this is not the major focus of this study, follow‐up analyses which include environmental viruses and bacteriophages not limited to those linked with health or disease in mammals might be better in deciphering the impact of ecological variation regarding the virome rather than pathogenic virus, examining associations between variation in viral diversity and host fitness or testing interactions between viral communities and other components of the host microbiome. (iii) The abundance and community composition of pathogens detected in domestic cats were slightly influenced by specific host behaviors such as vaccination. Future research could focus on selecting disease models based on clinical information, including vaccination status. (iv) Our stringent statistical tests and correction for multiple comparisons may have resulted in low(er) power and potential false-negative results. It is possible that other microbial taxa are differentially abundant or prevalent with age, in addition to those reported here. (v) Comparison of the healthy and diseased group would provide more specific infectomic dynamics such as contribution of each pathogen to specific symptoms. But due to the sparseness of ascitic fluid in healthy cats, sampling would be hardly feasible. Note that we failed to include ascitic fluid in our study, the healthy group from blood would lack reference and comparison. Future research sampling of swabs from the respiratory and digestive tract of diseased and healthy cats would be informative to understand pathogen contribution to a specific disease.

Despite these limitations, our study showed the capacity of meta-transcriptomics to reveal host *in vivo* transcription to discover the total pathogen array in a single assay. This approach allowed us to accurately assess pathogen abundance in diseased models and explore their genomic evolutionary details, enabling the identification of potential pathogens and the evaluation of their threat to human health. Additionally, we presented a comprehensive framework that combines meta-transcriptomics with experimental approaches to investigate the effects of environmental and host variables on the spread of infectious diseases. Specifically, we investigated the total infectome associated with diseased cats in China, revealing a diverse array of viruses, bacteria, and eukaryotic pathogens. While most of these pathogens are well-known cat pathogens, we identified a divergent species within the genus *Rickettsia* associated with self-limited disease in cats, as well as potential novel clinical outcomes associated with *M. aubagnense* in cats. In addition, our findings revealed that most of the cat diseases examined were better explained by the presence of co-infection with multiple pathogens rather than infection with a single pathogen. We concluded that altered demographic structure and ecological variables may have a cascading effect on the diverse dynamic of the infectome. More importantly, we showed the value of linking the modern influx of meta-transcriptomics with comparative ecology and demography and utilizing it to affirm that the ecological and demographic variation impacts not only the single-pathogen model but also the total infectome.

## Data Availability

All shotgun sequencing files have been deposited in the China National GeneBank DataBase (CNGBdb) under the accession number CNP0004735. All sequencing files were deposited in CNGBdb under the sequencing accession numbers N_AAAECY010000000 to N_AAAEDH010000000 (https://db.cngb.org/). Any remaining data can be acquired in the supplemental files or by request to the corresponding authors.

## SUPPLEMENTAL MATERIAL

Supplemental material msystems.00636-24-s0001.docx Supplemental methods; Fig. S1 to S7.

Supplemental tables msystems.00636-24-s0002.xlsx Tables S1 to S5.

ASM does not own the copyrights to Supplemental Material that may be linked to, or accessed through, an article. The authors have granted ASM a non-exclusive, world-wide license to publish the Supplemental Material files. Please contact the corresponding author directly for reuse.
