# Supplementary material for: Demographic and zoological drivers of infectome diversity in companion cats with ascites
Source: mSystems. 2024 Aug 9;9(9):e00636-24. doi: 10.1128/msystems.00636-24 (PMC11406987; doi:10.1128/msystems.00636-24)
Supplement: Supplemental material — Supplemental methods; Fig. S1 to S7. [file msystems.00636-24-s0001.docx]

#### Demographic and zoological drivers of infectome diversity in companion cats

**Summary:** The Supplementary information consists of a supplementary method, 7 figure legends, and 5 table notes.

### Supplementary Method

### Sensitive test using RT-qPCR

The abundance data was modeled with specific CT values to evaluate the rationality of the quantitative index from our transcriptomic data (i.e., RPM). To estimate the CT values of specific pathogens in the samples, specific probe and primers were designed to employ RT-qPCR assays on FIPV (Supplementary Table 1). RNA was first reverse transcribed to generate cDNA using the M-MLV kit (TaKaRa, Dalian China), and the cDNA was amplified using TaqPath ProAmp Master Mix (Applied Biosystems, California USA). Ct values generated from the qPCR were compared with the RPM value using the linear regression model.

### Supplementary Table 1 FIPV primers and probe for RT-qPCR

| Primer | Nucleotide | Region |
| --- | --- | --- |
| Forward | 5’-GATTTGATTTGGCAATGCTAGATTT-3’ | 7b gene |
| Reverse | 5’-ACCAATCACTAGATCCAGACGTTAGCT-3’ | 7b gene |
| Probe | FAM-TCCATTGTTGGCTCGTCATAGCGGA-TAMRA | 7b gene |

### Metadata variables

For hospitalized cats, we meticulously recorded environmental and demographic metrics, including sex, age (in months), landscape metrics of habitat suitability (precipitation, humidity, coordinates, and temperature), and detailed clinical symptoms (refer to Table S5). Specifically, clinical interviews with cat hosts conducted by veterinary professionals provided data on cat measures such as age and sex. Daily climate metrics for the sampled months were obtained from the local Climate Bureau at the city level and then normalized as monthly temperature per sample [°C], monthly precipitation per sample [mm], and monthly average humidity per sample [%]. In the case of temperature, precipitation, and humidity measures for stray cats, a similar approach was employed for collecting climate metadata, except for population density. To determine the population density of stray cats, we conducted a custom questionnaire survey to count the number of stray cats within a one-km^2^ scale around each sampling hospital. This approach aimed to explore the effects of spatial community size of stray cats on pathogen diversity. For the ages of stray cats, we determined by examining the level of fusion of the phalangeal epiphyses (Anthony, 1988).

### Supplementary Figures


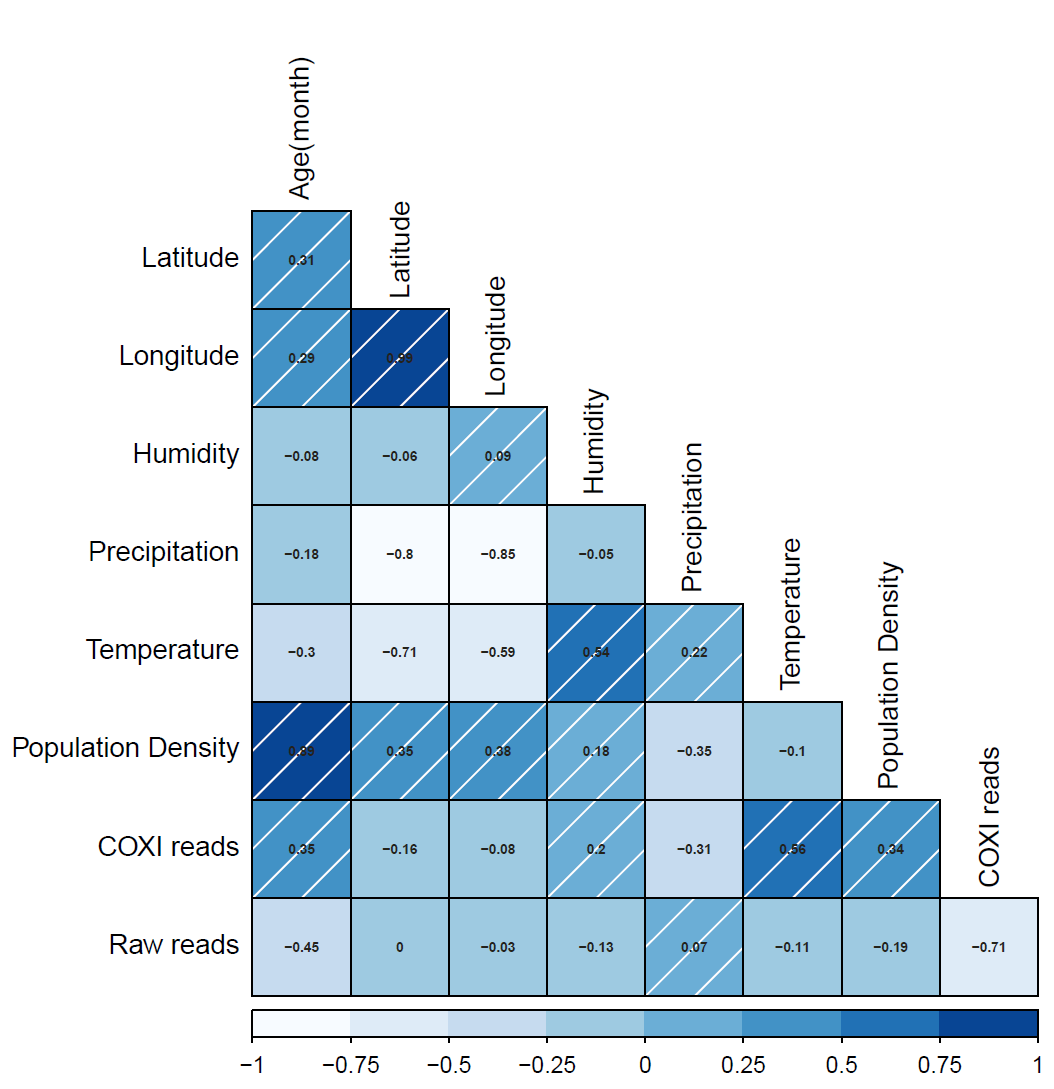


**Figure S1**: Pearson correlation analysis of pairwise explanatory variables, with p<0.05 shown as shading. For reducing overfitting, we removed the variables with r>0.7.


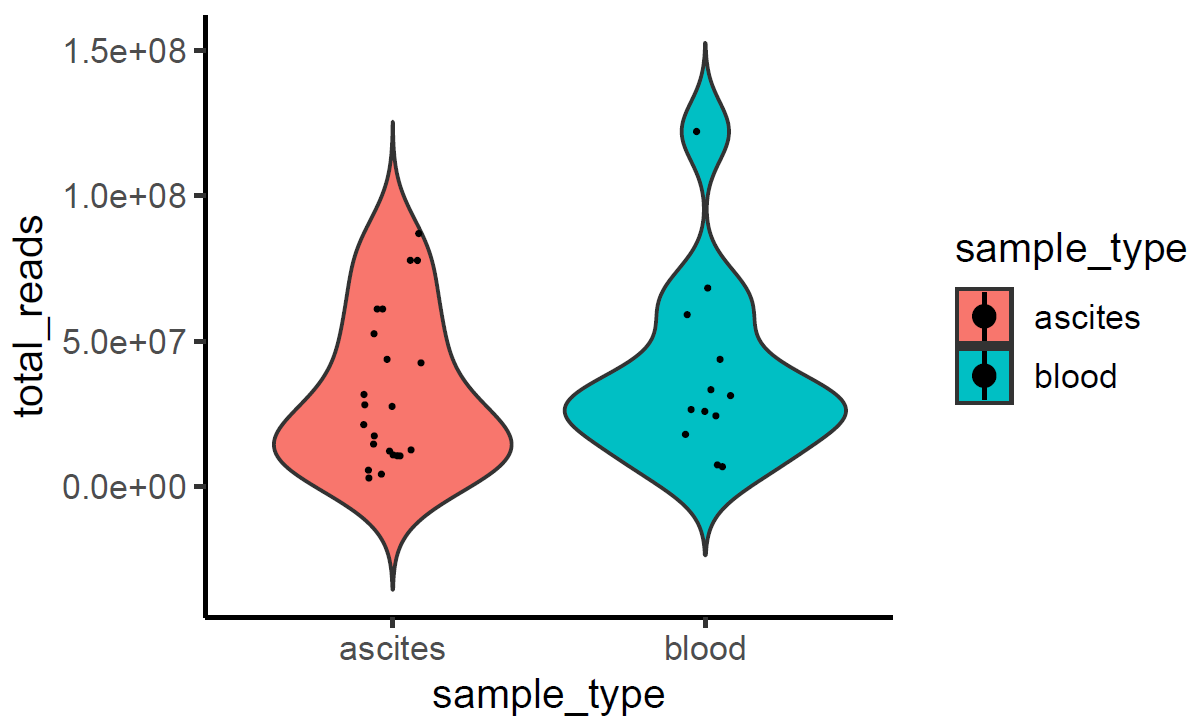


**Figure S2**: Comparison of total reads numbers of different types of samples.


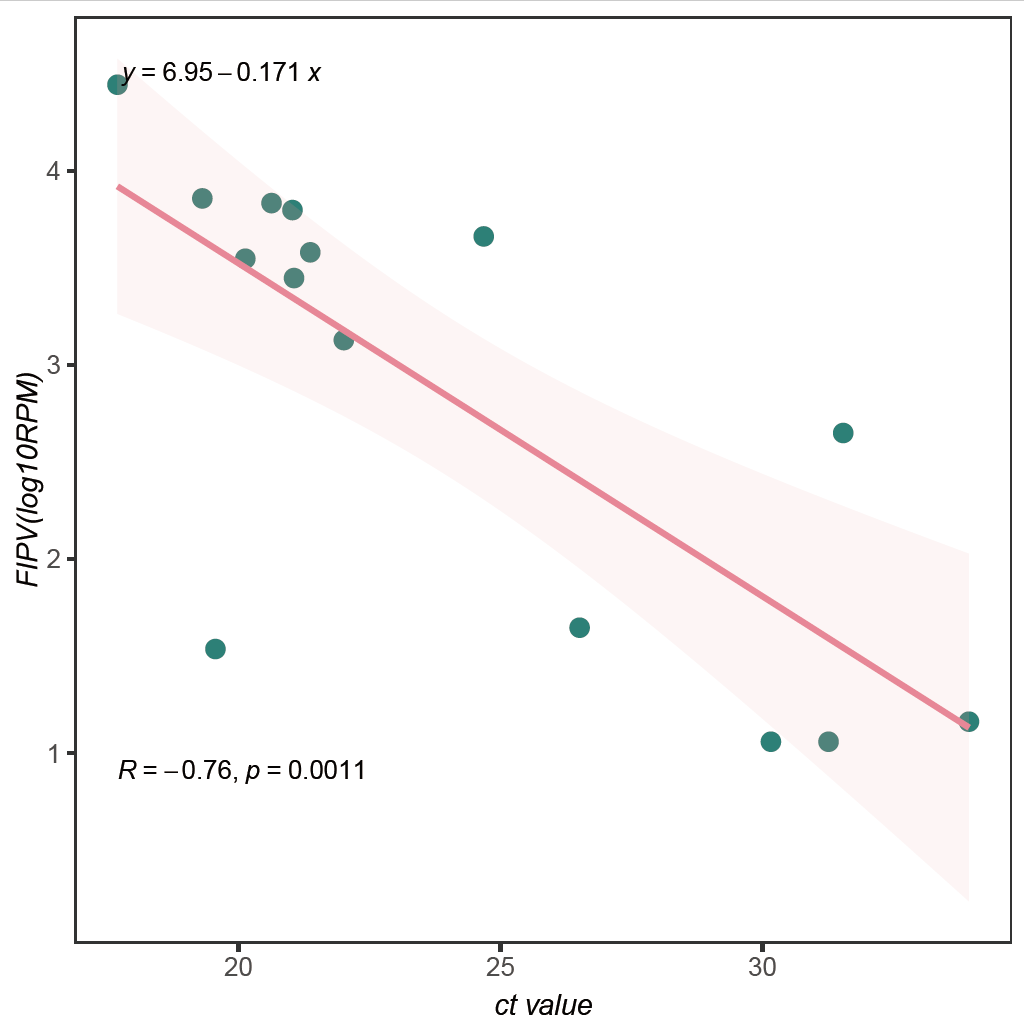


**Figure S3**: Correlation analysis between the viral loads (represented by CT value) and the abundance using three representative pathogens. The abundance (RPM) is in log10 scale (y-axis). The viral loads in-vivo were represented by the CT values. The pearson’s correlation coefficient (pearson r) between log10RPM and CT value is computed (p<0.05). Equations derived from linear modeling are given in the panel. 95% confidence intervals for the linear model fit are shown by colored ribbons.


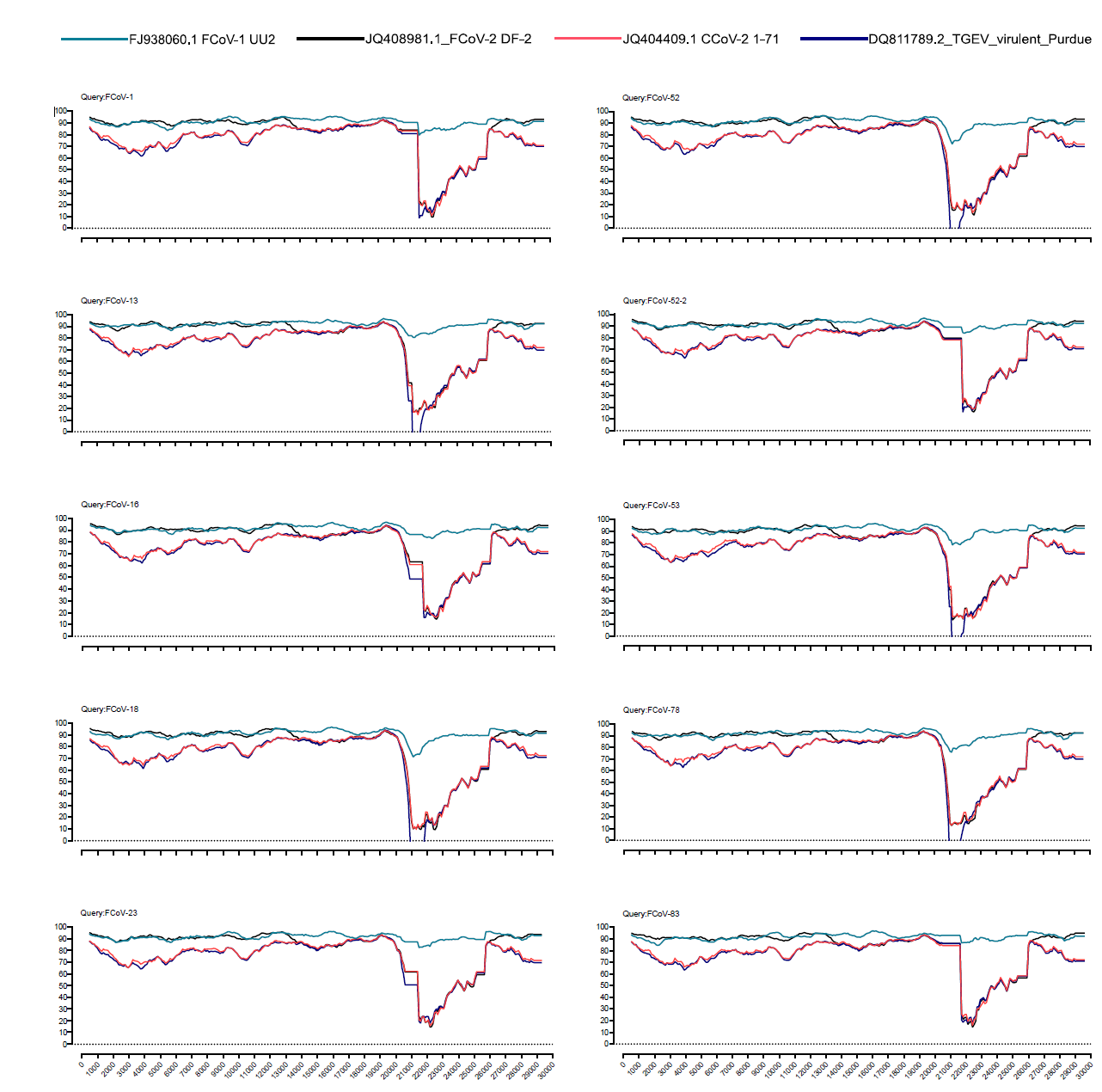


**Figure S4**: Recombination of all FCoVs identified here at the whole genome scale with CCoV-II, FCoV-I, FCoV-II, and TGEV.


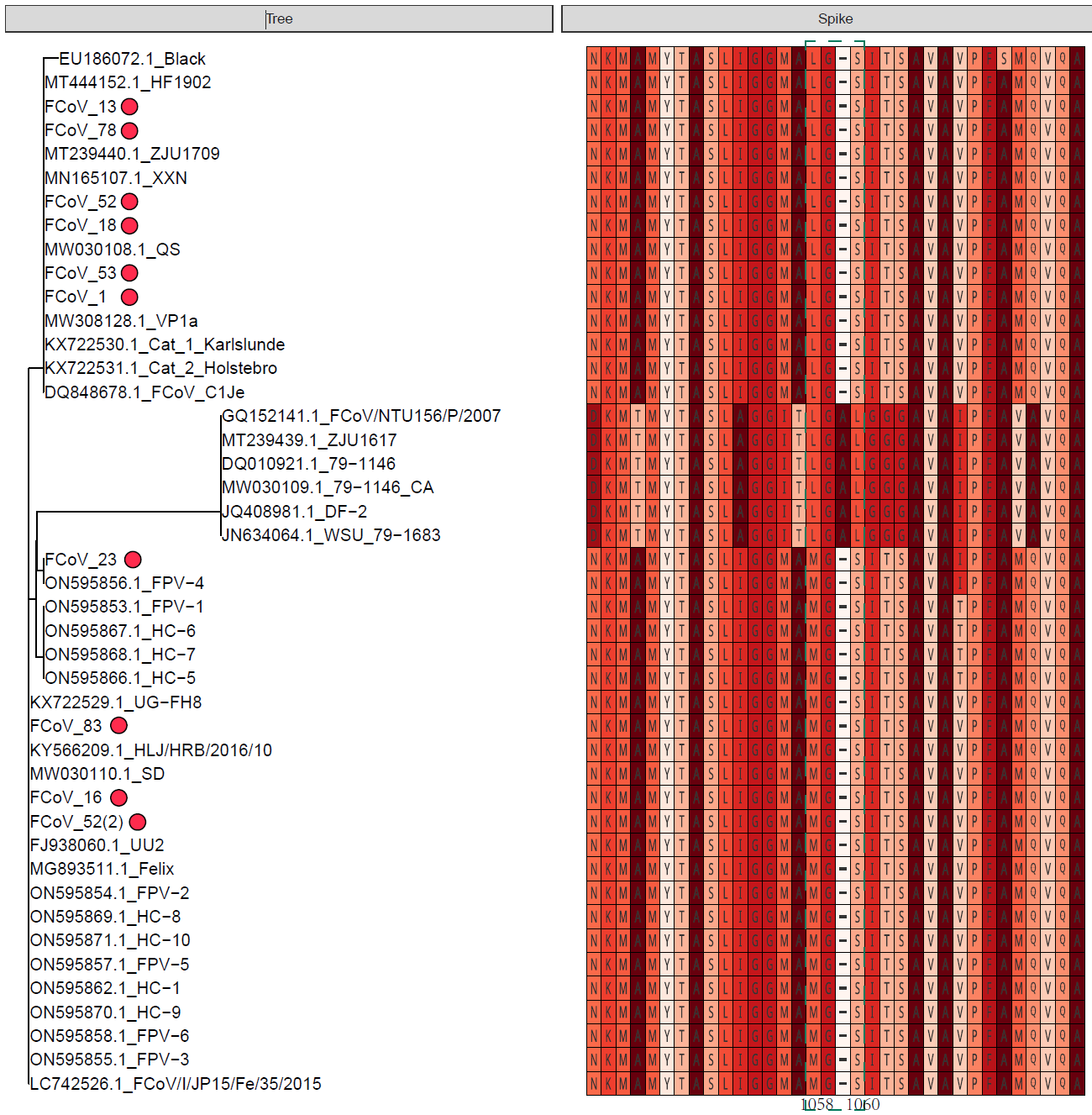


**Figure S5**: Maximum-likelihood phylogenetic analysis of partial amino acid in the spike gene with 1 000 replicates. The isolates identified in this study were marked with red dots at the tips (panel left). Key mutation of amino acid in the 1058 and 1060 sites (panel right).


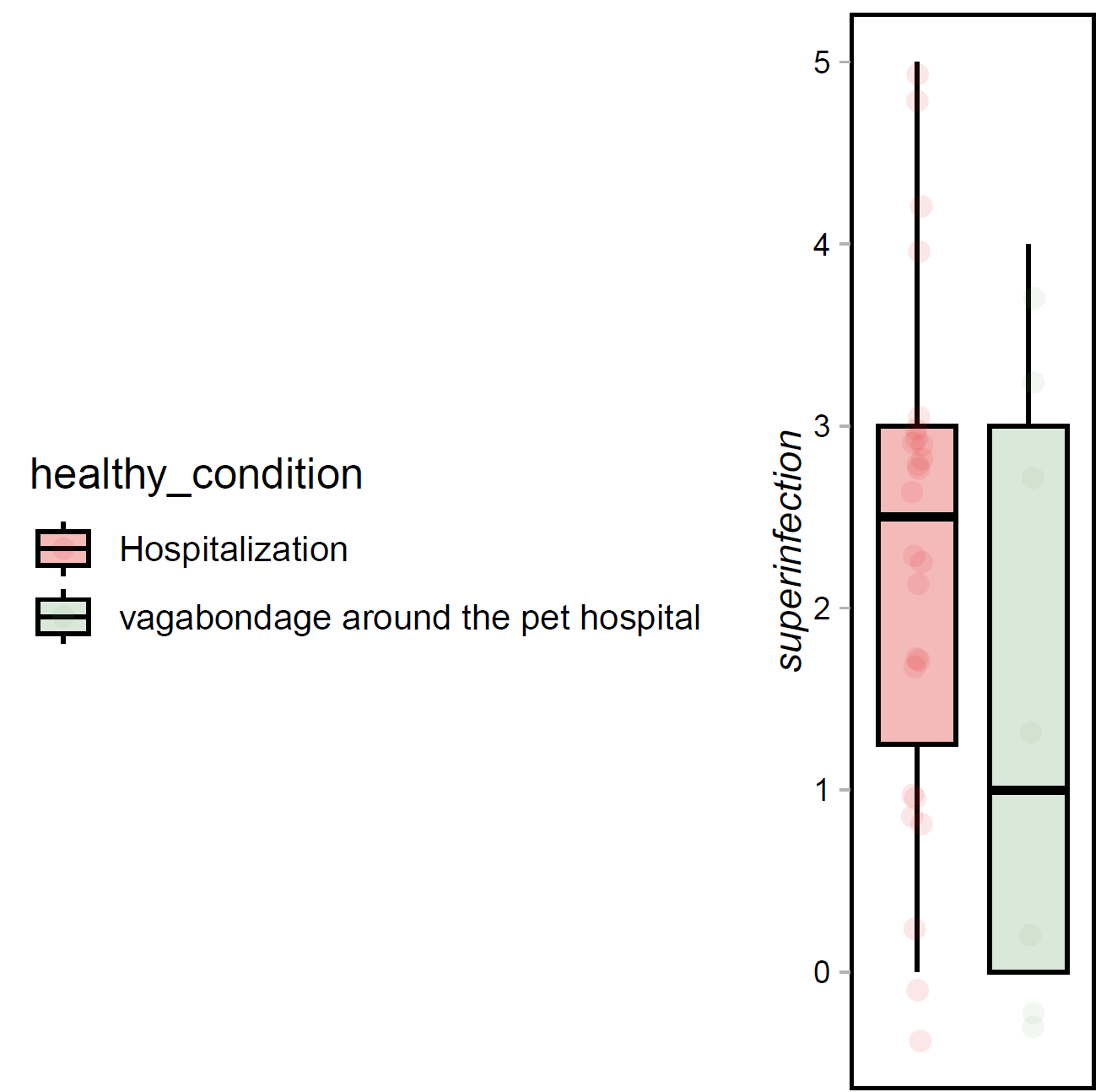


**Figure S6**: Comparison of superinfection numbers in stray cats and hospitalized cats.


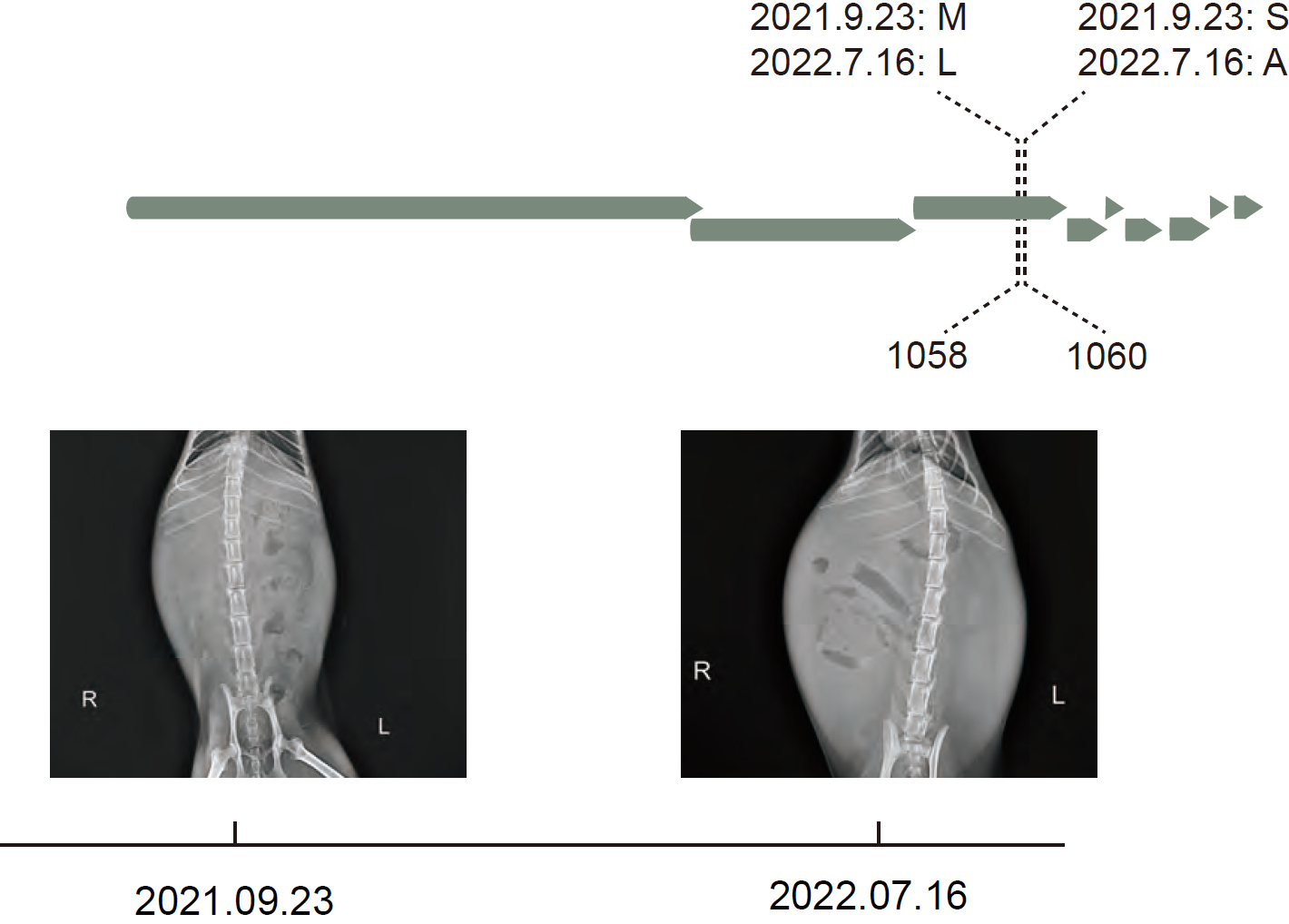


**Figure S7**: Continuous monitoring of a case of ascites hyperplasia whose etiology is the amino acid mutation of spike gene (FIPV) in 1058 and 1060. Specifically, the cat was diagnosed with suspected ascites hyperplasia and took the CT scanning on Nov 23, 2021. But the FIPV detection showed negative using a commercial FIPV kit, without marker site mutations in the amino acid position of 1058 and 1060. However, we detected the marker mutations in the 1058 and 1060 sites on Jul 16, 2022, with overt symptoms of ascites hyperplasia.

### Table notes:

**Table S1:** Table showing sample collection, pathogen abundance, and clinical signs.

**Table S2:** Multivariate PERMANOVA testing the explanatory dissimilarities between stray and hospitalized cats.

**Table S3:** Multivariate PERMANOVA testing the explanatory dissimilarities between ascites and blood samples.

**Table S4:** 95% confidence set of GLMs for Shannon values of total infectome. Model averaging of the best-fit model presented was used to estimate effect sizes and confidence intervals for each explanatory variable.

**Table S5:** Ecological and demographic metadata of clinical samples.

### Reference

Anthony, J., 1988: Veterinary dentistry. Can Vet J, 29, 685-688.
